# Thalamic Foxp2 regulates output connectivity and sensory-motor impairments in a model of Huntington’s Disease

**DOI:** 10.1007/s00018-023-05015-z

**Published:** 2023-11-21

**Authors:** Ened Rodríguez-Urgellés, Diana Casas-Torremocha, Anna Sancho-Balsells, Iván Ballasch, Esther García-García, Lluis Miquel-Rio, Arnau Manasanch, Ignacio del Castillo, Wanqi Chen, Anika Pupak, Veronica Brito, Daniel Tornero, Manuel J. Rodríguez, Analia Bortolozzi, Maria V. Sanchez-Vives, Albert Giralt, Jordi Alberch

**Affiliations:** 1 Facultat de Medicina, Departament de Biomedicina, Institut de Neurociències, Universitat de Barcelona, 08036 Barcelona, Spain; 2 Institut d’Investigacions Biomèdiques August Pi I Sunyer (IDIBAPS), Barcelona, Spain; 3 Centro de Investigación Biomédica en Red Sobre Enfermedades Neurodegenerativas (CIBERNED), Madrid, Spain; 4 Institute of Biomedical Research of Barcelona (IIBB), Spanish National Research Council (CSIC), 08036 Barcelona, Spain; 5 Biomedical Research Networking Center for Mental Health (CIBERSAM), Institute of Health Carlos III (ISCIII), 28029 Madrid, Spain; 6 Faculty of Medicine and Health Science, Production and Validation Center of Advanced Therapies (Creatio), University of Barcelona, 08036 Barcelona, Spain; 7 Institució Catalana de Recerca I Estudis Avançats (ICREA), Barcelona, Spain

**Keywords:** Thalamus, Dendritic spines, Electrophysiology, Basal ganglia, Sensory information, Motor coordination, R6/1

## Abstract

**Background:**

Huntington's Disease (HD) is a disorder that affects body movements. Altered glutamatergic innervation of the striatum is a major hallmark of the disease. Approximately 30% of those glutamatergic inputs come from thalamic nuclei. Foxp2 is a transcription factor involved in cell differentiation and reported low in patients with HD. However, the role of the Foxp2 in the thalamus in HD remains unexplored.

**Methods:**

We used two different mouse models of HD, the R6/1 and the HdhQ111 mice, to demonstrate a consistent thalamic Foxp2 reduction in the context of HD. We used in vivo electrophysiological recordings, microdialysis in behaving mice and rabies virus-based monosynaptic tracing to study thalamo-striatal and thalamo-cortical synaptic connectivity in R6/1 mice. Micro-structural synaptic plasticity was also evaluated in the striatum and cortex of R6/1 mice. We over-expressed Foxp2 in the thalamus of R6/1 mice or reduced Foxp2 in the thalamus of wild type mice to evaluate its role in sensory and motor skills deficiencies, as well as thalamo-striatal and thalamo-cortical connectivity in such mouse models.

**Results:**

Here, we demonstrate in a HD mouse model a clear and early thalamo-striatal aberrant connectivity associated with a reduction of thalamic Foxp2 levels. Recovering thalamic Foxp2 levels in the mouse rescued motor coordination and sensory skills concomitant with an amelioration of neuropathological features and with a repair of the structural and functional connectivity through a restoration of neurotransmitter release. In addition, reduction of thalamic Foxp2 levels in wild type mice induced HD-like phenotypes.

**Conclusions:**

In conclusion, we show that a novel identified thalamic Foxp2 dysregulation alters basal ganglia circuits implicated in the pathophysiology of HD.

**Supplementary Information:**

The online version contains supplementary material available at 10.1007/s00018-023-05015-z.

## Background

Huntington's disease (HD) is an autosomal dominant inherited neurodegenerative disorder characterized by motor coordination alterations [1], and caused by a pathological CAG repeat expansions in the human huntingtin (htt) gene on chromosome 4p16.3 [2, 3]. Medium-spiny projection neurons (MSNs) of the caudate nucleus and putamen (striatum) are the most vulnerable cell types suffering progressive neurodegeneration, which eventually lead to death [4]. It has been demonstrated that this severe striatal degeneration is induced or initiated, at least in part, by aberrant excitotoxic processes from afferent terminals [4, 5]. The striatum receives its main glutamatergic inputs from the pyramidal cells located in deep cortical layers (up to a ~ 60 to 70%), whereas the rest of the glutamatergic inputs come from thalamic nuclei (up to a ~ 30 to 40%) [6]. Although the cortico-striatal aberrant connectivity has been largely studied in the HD context, the thalamo-striatal connectivity, including the Centromedial-Parafascicular Nuclear Complex (intralaminar nuclei) and the Ventral Posteromedial Nucleus (VPM) [7–9], has been poorly addressed.

Degeneration of the thalamus and some of its nuclei in HD patients has been reported elsewhere [10–12]. In human studies using positron emission tomography, it has been identified an enhanced thalamic activation in preclinical HD subjects during the performance of a motor learning task [14, 15]. In this line, the thalamo-striatal pathway shows early changes in thalamo-striatal afferent connectivity preceding MSNs neuropathology in an HD mouse model [5, 16], suggesting that loss or alterations of the thalamo-striatal terminals could significantly contribute to HD-associated deficits [17, 18]. Furthermore, previous in vitro co-culture works reveal that thalamo-striatal synapses in MSNs are significantly altered from early stages in HD mouse models, including altered AMPA excitability and increased probability of vesicular glutamate release that might emerge as a possible compensatory mechanism for the loss of thalamo-striatal inputs [19, 20]. Despite these initial descriptive works, the role of thalamic alterations in the progression of HD has been scarcely addressed [21, 22] but its origin and physiological role or the underlying molecular mechanisms remain to be elucidated. In this sense, a crucial molecule that could mediate such alterations is Foxp2.

Foxp2 belongs to a family of transcription factors [23] called FOX proteins initially described to be implicated in the regulation of cell growth and differentiation as well as embryogenesis [24]. Subsequent studies have found that FOXP2 is decreased in the putamen of postmortem samples from HD patients and that striatal over-expression of Foxp2 rescues some motor coordination deficits in a HD mouse model at late stages of the disease [25]. We have recently described in mouse models of HD that changes in Foxp2 expression and function in MSNs from postnatal day 15 onwards regulate very early molecular changes and synaptic plasticity impairments associated with the earliest psychiatric-like phenotypes of those models [26]. However, the role of Foxp2 alterations in HD could be more global by playing, for example, a role in the thalamo-striatal connections. Accordingly, Foxp2 is mainly expressed not only in MSNs but also in thalamic neurons during embryogenesis and during adulthood [27, 28]. Given the role of Foxp2 in the synapse formation and plasticity as well as in the neurite outgrowth and neuronal circuits formation and maintenance [27, 29–34], it is conceivable that the early and severe reduction that we previously observed in the HD striatum and cortex [26] is also taking place in the thalamus. Thus, we hypothesize that such Foxp2 alterations might have the potential to play a key role in the aberrant thalamo-striatal connectivity leading to the development of HD symptoms.

In the present work, we show that thalamo-striatal connections are early affected together with a severe reduction of Foxp2 levels in the thalamus in exon-1 and knock-in mouse models. The rescue of Foxp2 levels in the R6/1 thalamus prevented spine loss in the striatum and in the sensory cortex, corrected motor coordination and sensorial alterations and amended specific thalamo-striatal and thalamo-cortical synaptic activity deficiencies which, in turn, could be related with an alteration GABA and glutamate release. Finally, reducing Foxp2 levels in the ventrobasal thalamus of wild-type (WT) mice was enough to induce an HD-like phenotype supporting the relevance of thalamic Foxp2 in the pathophysiology of HD.

## Methods

### Animals

Transgenic R6/1 mice [35] expressing the N-terminal exon-1 fragment of mHtt containing 115 CAG repeats, were used. HdhQ7 WT mice with 7 CAG repeats and HdhQ111 knock-in mice (KI), with targeted insertion of 109 CAG repeats that extends the glutamine segment in murine huntingtin to 111 residues, were also used [36]. Genotypes were determined by polymerase chain reaction (PCR) from ear biopsy. Microchips were implanted under the mice skin providing information about their birth, location, and genotype. All mice were housed in numerical birth order in a room kept at 19–22 °C and 40–60% humidity under a 12:12 light/dark cycle with access to water and food ad libitum. All experiments were conducted exclusively with male mice. WT littermates were used as a control group. Standard animal procedures were approved by the animal experimentation Ethics Committee of the Universitat de Barcelona (274/18) and Generalitat de Catalunya (10/20), in agreement with the Spanish (RD53/2013) and European (2010/63/UE) regulations for the care and use of laboratory animals.

### Rabies virus-based monosynaptic tracing

To target the pre-synaptic inputs received by the striatum, we utilized a two-virus system adapted from a previous protocol [37]. Both viruses, the helper lentivirus containing the pBOB-Syn-hisGFP-TVA-rabiesG plasmid (10% dilution from titre: 9 × 10^7^ TU/ml) and the pseudo-typed rabies virus EnvA-ΔG-mCherry (5% dilution from titre: 25 × 10^6^ TU/ml) were provided by Dr. Malin Parmar from Lund University, Sweden. Stereotaxic surgery was performed in 4-week-old and 10-week-old R6/1 male mice and WT littermates. Mice were anesthetized with 3.5% isoflurane in an induction chamber. Then, mice were fixed in the stereotaxic apparatus being anesthetic status maintained with 1.5% isoflurane. A dose of 2 mg/kg of analgesic Metacam® was injected subcutaneously. 1 μl of viral vectors was injected targeting dorsal striatum following coordinates relative to Bregma (anteroposterior and lateral) and from skull (dorsoventral): AP: + 0.5 mm; L: + 1.8 mm and DV: – 2.55 mm were used for the 4-week-old animals. For the 10-week-old animals the coordinates were: AP: + 0.8 mm; L: + 2 mm and DV: – 2.7 mm. Viral vectors were injected with a 5 μl Hamilton Neuros syringe at an infusion rate of 100ηl/min. The needle was left in place for 5 min to ensure complete diffusion of the virus. After 7 days, animals were conducted to the second stereotaxic surgery and then infected with the pseudo-typed rabies virus, using an angle of 45º to avoid undesired infection of cortical neurons due to residual virus in the needle track. The following coordinates relative to Bregma (anteroposterior and lateral) and from skull (dorsoventral): AP: – 0.5 mm; L: + 3.45 mm and DV: – 2 mm were used for the 4-week-old animals. For the 10-week-old animals the coordinates were relative to Bregma (anteroposterior and lateral) and from skull (dorsoventral): AP: – 0.8 mm; L: + 3.65 mm and DV: – 2.35 mm. Seven days later, mice were intracardially perfused with 4% paraformaldehyde. After perfusion, the brain was isolated and post fixed with 4% paraformaldehyde overnight at 4 °C. The day after, brains were transferred to a 30% sucrose solution in PBS with 0.02% Sodium Azide, and kept at 4 °C. Then, 40 μm coronal brain sections were prepared using a microtome (Leica SM2010 R) and stored in a cryoprotectant anti-freeze solution (30% glycerol, 30% ethylene glycol and 15% Tris–HCl) at − 20 °C.

Brain sections were immunostained with antibodies against mCherry and GFP and mounted on glass slides. Slides were scanned with an Olympus BX51 microscope (Olympus, Ballerup, Denmark). Sections were analyzed using the Optical Fractionator technique and stereology software from visiopharm (version 7.0.3.3313). Primary somatosensory and motor cortices (S1-M1) and thalamic neuronal inputs to striatum were evaluated with 40 × objective by counting traced cells (expressing only mCherry) that fell within the different brain areas delineated based on DAPI staining and brain mouse atlas. Striatal starter neurons were evaluated by counting cells infected with both viruses (expressing nuclear GFP and cytoplasmic mCherry) that fell within the striatum. For each animal, the number of cortical and thalamic traced neurons was normalized dividing by the number of striatal starter neurons.

### Tissue preparation and immunofluorescence

Mice were euthanized by cervical dislocation. Brains were removed and fixed for 72 h with paraformaldehyde 4% (PFA), and then kept in PBS with 0.02% Sodium Azide at 4 °C until use. Coronal sections (40-μm) of the brain were obtained using a vibratome (Leica VT 1000S) and kept in cryoprotectant anti-freeze solution at − 20 °C until use. Free-floating brain sections were rinsed twice in PBS for min and incubated 2 times for 15 min each with 50 mM NH_4_Cl to reduce aldehyde-induced tissue autofluorescence. Sections were permeabilized twice for 10 min each with PBS containing 0.5% Triton X-100. Thereafter, sections were blocked with PBS containing 0.3% Triton X-100, 0.2% Sodium Azide and 5% normal donkey serum/goat serum (Pierce Biotechnology, Rockford, IL) for 2 h at room temperature. Then, sections were incubated overnight at 4 °C in the presence of primary antibody in PBS-T: rabbit Foxp2 antibody (1:100, Abcam #ab16046, Cambrige, UK), DARPP-32 (1:500; BD Transduction # 611,520, New Jersey, USA); parvalbumin (1:1000, Swant #PV27, Burgdorf, Switzerland), ChAT (1:500; Chemicon #AB144P, Burlington, USA), VGlut2 (1:1000, Millipore #AB2251-I, Burlington, USA) and PSD-95 (1:250, Cell Signaling #3450S, Danvers, USA). Sections were then washed three times and incubated for 2 h at room temperature with fluorescent secondary antibodies: Cy3 goat anti-rabbit (1:200) and/or AlexaFluor 488 donkey anti-mouse (1:200; both from Jackson ImmunoResearch, West Grove, PA, USA). No signal was detected in control sections incubated in the absence of the primary antibody.

### Confocal imaging and analysis

Immunostained tissue sections (40-μm thick) containing ventrobasal thalamus were imaged using a Leica Confocal SP5-II (20× or 40× numerical aperture lens, 5 × digital zoom, 1-Airy unit pinhole). At least two slices per mouse, were analyzed, and up to two representative striatum images were obtained from each slice. Four frames were averaged per z-step throughout the study. Confocal z-stacks were taken at 1024 × 1024-pixel resolution every 2 μm. Foxp2 nuclei integrated optical density was quantified with NIH ImageJ freeware (Wayne Rasband, NIH). Colocalization of VGlut2 and PSD-95 double-positive puncta were quantified as previously described [38].

### Western blot

Quantification of protein fraction was performed using the Detergent-Compatible Protein Assay (Bio-Rad, Hercules, CA, USA). Protein extracts (20 μg) were denatured in 62.5 mM Tris–HCl (pH 6.8), 2% Sodium dodecyl sulfate (SDS), 10% glycerol, 140 mM b-mercaptoethanol, and 0.1% bromophenol blue and heated at 100 °C for 5 min. Protein extracts were resolved in denaturing SDS–polyacrylamide gel electrophoresis (SDS-PAGE), with variable polyacrylamide concentration depending on the molecular weight of the protein of interest, at 35 mA/gel over 1 h. The Precision Plus ProteinTM Dual Color ladder (Bio-Rad) was loaded along with the protein samples to properly identify the protein of interest. Afterwards, proteins were transferred to a nitrocellulose membrane (Whatman Schleicher & Schuell, Keene, NH, USA) during 1.5 h at 90 V at 4 °C. Membranes were rinsed with Tris-buffered saline, 0.1% Tween 20 (TBS-T) to remove staining. Non-specific protein binding sites were blocked during 1 h incubation in blocking solution containing 10% non-fat powdered milk in TBS-T (50 mM Tris–HCl, 150 mM NaCl, pH 7.4, 0.05% Tween 20). Membranes were rinsed 3 times during 10 min in TBS-T and immunoblotted overnight at 4 °C with the primary antibody rabbit anti-Foxp2 (1:1000, Abcam #ab16046, Cambrige, UK). Membranes were then rinsed 3 times for 10 min each with TBS-T and incubated with the proper horseradish peroxidase-conjugated secondary antibody for 1 h at room temperature.

### Intra-thalamic injections of adeno-associated viruses

Stereotaxic surgery was carried out in 10–12-week-old WT mice by ketamine–xylazine-induced anesthesia. Mice were anesthetized with 3.5% isoflurane in 100% oxygen in an induction chamber. Then, fixed in the stereotaxic apparatus anesthetic status was maintained with 1.5% isoflurane. Mouse head was shaved and cleaned with ethanol. Then iodine and local anesthesia were applied (lidocaine 2.5% and prilocaine 2.5% EMLA®, AstraZeneca), and a dose of 2 mg/kg of analgesic Metacam^®^ was injected subcutaneously. 500ηl of viral vector were injected in each hemisphere targeted bilaterally the thalamus. The following coordinates relative to Bregma (anteroposterior and lateral) and from skull (dorsoventral) were used to target the ventrobasal thalamus: AP: – 1.7 mm; L: ± 1.5 mm and DV: – 3.5 mm. Viral vectors (AAV9-CamKIIa-eGFP-2A-mFoxp2-WPRE (Vector Biolabs, Pennsylvania, USA), AAV5-CamKIIa-eGFP-2A-WPRE (UNC Vector Cre, North Carolina, USA), AAV8-mCherry-U6-mFoxp2-shRNA (#shAAV-259,597, Vector Biolabs, Pennsylvania, USA) and AAV5-mCherry-U6-scrmb-shRNA (#1781, Vector Biolabs, Pennsylvania, USA); titers: 7–8.5 × 10^12^) were injected with a 5 μl Hamilton syringe at an infusion rate of 100ηl/min. We used the CamKIIa promoter for our AAVs since we had previous very good viral transduction rates [26] and since CamKIIa is highly expressed in the thalamus [39]. The needle was left in place for 5 min to ensure complete diffusion of the AAVs. Mice were return to their home cage after fully recovery. All mice subjected to surgery that survived and showed no ethical and healthy problems (such as head inclination or > 15% of body weight loss) were the ones used for behavioural characterization.

### Novel whisker-dependent texture discrimination test

Novel whisker-dependent texture discrimination test was conducted as previously reported [40]. It consisted of two consecutive days of habituation in the open field, and one day for testing divided in two sessions: training and testing session. A gray open-top square arena (40 cm × 40 cm long; with 30 cm high walls) was placed in a room with dim light (20–25 lx). On testing day, for the first session mice were placed in the testing apparatus with two identically smooth-textured objects at the center of the arena. Mice were allowed to explore the objects for 5 min and then removed and held in a cage for 5 min. Before the second session, the two objects in the arena were replaced with a third one identically textured object and a novel object with a different texture. Mice were then placed back into the arena for the second session, the test phase, and allowed to explore for 1 min. The amount of time mice spent actively investigating the objects was recorded. Animal tracking was recorded via a CCD camera. Image data acquisition and analysis were performed automatically using Panlab SMART Video Tracking System (3.0).

### Accelerating rotarod

The accelerating rotarod training procedure (ARTP) was conducted as previously described [41]. To evaluate mouse motor learning and performance, mice were tested on the accelerating rotarod over 2 days. Animals were placed on a horizontal rotating rod (30 mm diameter) with an increasing gradually speed (4–40 RPM) over 5 min. Latency to fall was recorded as the time mice spent in the rod before falling. The testing session consisted of 3 trials a day for 2 consecutive days, with a 1 h inter-trial interval.

### Golgi and Nissl staining and dendritic spine analysis and volumes estimations

Fresh brain hemispheres were processed following the Golgi–Cox method as described elsewhere [42]. Secondary dendrites from stellate neurons in the layer IV of the somatosensorial cortex and in the ventrobasal thalamus were photographed, with a maximum of two–three dendrites per neuron and from at least 3 slices per animal. Z-stacks from 0.2 μm sections were obtained in bright field at 63 × resolution on a Widefield AF6000 Monochrome Camera Leica Microscope. Images were analyzed with the ImageJ software. Secondary dendritic segments (> 20 μm long) were selected and traced. The total number of spines was obtained using the cell counter tool from ImageJ. Dendritic spine density was obtained after dividing the number of spines by the length of the segment (n° spines/μm). At least 60 dendrites in cortical neurons and 20 dendrites in thalamic neurons per group from at least four mice per genotype were counted. The head diameter and the spine length were measured as previously described [42]. Nissl staining protocol was performed as previously reported [43]. Brain regions (striatum and ventrobasal thalamus) volumes were estimated using the Cavalieri method as previously described [44].

### Unbiased stereology

Striatal and thalamic volumes of Cytochrome C Oxidase (see below for further details about the immunohistochemical protocol) and Nissl-stained brain sections were estimated using the Cavalieri method as previously described [44].

### Surgical procedures for in vivo electrophysiology

Anesthesia was induced with an intraperitoneal injection of urethane (1.6 g/kg) in 16-week-old WT and R6/1 mice. During recording sessions, the level of anesthesia was monitored by the presence of delta frequency waves (1–4 Hz) of high amplitude (> 50 µV) in the local field potentials (LFP) as well as by the absence of both spontaneous whisker movements and pinch withdrawal reflex. Atropine (0.3 mg/kg), methylprednisolone (30 mg/kg), and mannitol (40 mg/kg) were administered subcutaneously to avoid respiratory secretions and edema. The animal was placed on a water-heated pad (RWD Life Science, China) set at 37 °C to keep body temperature stable, and the head was positioned in a rodent stereotaxic frame (SR-6 M, Narishige, Japan). Local anesthetic (lidocaine 1%) was applied to all skin incisions and a craniotomy was performed using mini-rongeurs over the left hemisphere from − 3.0 mm to + 3.0 mm relative to bregma and + 3.0 mm relative to midline. This broad craniotomy was selected to access a large area of the targeted hemisphere.

### Electrical stimulation in vivo

Electrical stimulation in the thalamus, with the aim of evoking responses in the striatum and the cortex, was applied by means of a stainless-steel bipolar electrode (210 μm spacing between tips; FHC Inc., USA), with a constant current isolated stimulator (DS3, Digitimer Ltd., UK) controlled by Spike2 software using a CED Power 1401 interface (Cambridge Electronic Design, UK). The tip of the stimulation electrode was stereotaxically positioned into the ventral posteromedial thalamic nucleus (VPM) following coordinates according to the Gaidi mouse atlas (AP: − 1.9 mm, LM: − 1.7 mm; DV: − 3.4 mm) in WT animals. These coordinates were adapted for R6/1 mice (AP: − 1.7 mm, LM: − 1.6 mm; DV: − 3.3 mm). For each protocol applied, 50 single square pulses (0.3 ms duration) were delivered at 0.1 Hz with intensity currents ranging from 40 to 320 µA (steps of 40 µA) to elicit thalamo-striatal responses and ranging from 20 to 160 µA (steps of 20 µA) to elicit thalamo-cortical responses.

### Electrophysiological recordings in vivo

Spontaneous and evoked local field potentials (LFP) were recorded in the striatum and the cortex. First, a tungsten microelectrode (2–4 MΩ; FHC Inc., USA) was lowered stereotaxically into the striatum following the coordinates AP: − 0.9 mm, LM: − 3.3 mm; DV: − 2.25 mm for WT mice and AP: − 0.8 mm, LM: − 3.2 mm; DV: − 2.25 mm for R6/1 mice. The tungsten microelectrode was previously coated with a fluorescent dye (Vybrant^®^ DiI or Vybrant^®^ DiO; Invitrogen) to enable the visualization and location of the tip under the microscope. A total of 500 s of spontaneous activity were recorded prior to the stimulation protocol, which was applied in increasing current intensities. When all the striatal recordings were completed, the electrode was slowly removed from the brain. Then, a 32-channel multielectrode array ([45]; 550 μm spacing between recording points) was positioned in the cortical surface covering the motor and the primary and secondary somatosensory areas. In the cortex, 500 s of spontaneous activity were recorded and then electrical stimulation was applied for all increasing current intensities. For both striatal and cortical recordings, the signal was amplified by 100 and high-pass filtered above 0.1 Hz (Multichannel Systems, GmbH), digitized at 5 kHz and fed into a computer via a digitizer interface (CED 1401 and Spike2 software, Cambridge Electronic Design, UK). Mice were sacrificed with an overdose of anesthetics (see below) immediately after recordings were completed.

### Data analysis from in vivo electrophysiological recordings

In the spontaneous activity for both striatal and cortical recordings, the detection of the transitions between silent (Down) and active (Up) states was performed using a z-score normalized multivariate time series, composed by the raw signal (LFP), a logarithmically-scaled estimation of 200–1500 Hz power of the LFP (the Multi-Unit Activity or MUA) [46], and the envelope of the variance of the gamma-filtered LFP [47]. For each LFP signal, a highly processed data time sequence was obtained resulting from the linear combination of the three-time sequences described above, using principal component analysis (PCA) to weigh the contribution of each of them. PCA was applied to the time series and a bimodal distribution originated from the projections over the first principal component, whose peaks corresponded to samples of activity belonging to Up or Down events. This bimodal distribution allowed us to select a threshold that optimally separated the two modes, enabling the creation of a binary signal that contained 1’s in those time samples where logMUA was over the threshold (Up state) and 0’s where below (Down state). A minimum duration of 80 ms for Up and Down state was set to avoid the detection of random signal fluctuations. Every detection was visually examined and validated to be included in the analysis. After Up and Down state detection, mean Up state durations were obtained [48]. The average firing rate for Up and Down states was computed as the mean value of the logMUA for all those time samples belonging to 1 and 0 respectively. The Power Spectral Density (PSD) was computed using Welch’s method from scipy (scipy.org, signal.welch) over the z-scored normalized LFP with a 2-s window and 0.1 Hz bin size.

Evoked potentials elicited by thalamic stimulation were extracted in the striatum and the cortex by calculating the average from responses to 50 stimuli. Given the complexity of the evoked responses and the difficulties in automating the detection of the positive and negative peaks of the generated waveforms, a customized Python pipeline was designed, with some small graphical widgets (ipywidgets) embedded in it. This pipeline allowed a semi-automatic detection of the peaks that was later validated by the experimenter. The mean of the peak latency, peak amplitude and the area under the curve were calculated from the evoked potentials. A baseline normalization was applied to all evoked responses such that the mean of all baselines was set to 0. The detection of the peaks was computed over the trial-averaged evoked responses for all intensities, according to the spatial coordinates of the recording array in the case of the cortical recordings. When performing the detection, the median was also displayed in case some outliers were observed. No significant differences between the mean and the medians were found. The first positive and negative peaks, if any, were detected. The transition times, considered as the points where either the positive or negative responses started to increase or decrease, were detected to compute the overall magnitude of the response (e.g., the area under the curve and peak amplitude). Peaks were only detected if they had an absolute amplitude value higher than 20 µV and occurred in the range of 3–50 ms after the stimulus onset. Then, the peak amplitude and the area under the curve (AUC) metrics were computed. The amplitude was taken as the absolute difference between the detected peak of the response and the first transition point from the same response. The AUC was computed as the absolute value of the integral of the response, whose boundaries were marked by the response’s transition points. In addition, long-lasting baseline z-score normalized evoked responses were also analyzed using a 600 ms post-stimuli time window. AUC and the standard deviation of the 32-channel-averaged evoked response were computed. Given the sample size and the comparison across only three groups in these experiments, we sought a balance between identifying true significant differences and the risk of false positives. We opted for the Benjamini-Hochberg [49] procedure to investigate the false discovery rate (FDR). The approach revealed an overall FDR of 0.125 across all our tests, suggesting that approximately 12.5% of the detected significant results might be false positives.

### Histological processing of mice from electrophysiological recordings in vivo

After electrophysiological recordings were completed, all animals were perfused transcardially, and brains processed for histology as described above. One of the series was used to analyze the location of the microelectrode in striatum coated with fluorescent dye. All nuclei were labeled with Bisbenzimide (Hoescht, Thermo Fisher Scientific; Waltham, MA, USA). The second series was stained using the cytochrome oxidase histochemistry protocol (CyO; [50]) to help cytoarchitectonic localization of both stimulation and recording electrodes. Both series were mounted onto gelatin-coated glass slides, air dried, dehydrated in graded ethanol, defatted in xylene and coverslipped with DePex (Serva). These sections were analyzed and photographed under an epifluorescence light microscope (Eclipse 600; Nikon).

### In vivo microdialysis

Extracellular glutamate and GABA concentration were measured by in vivo microdialysis as previously described [51, 52]. Briefly, one concentric dialysis probe (Cuprophan membrane; 6000 Da molecular weight cut-off; 1.5 mm long) was implanted in the striatum (AP, 0.5; LM, – 1.7; DV, – 4.5 in mm) of isoflurane-anesthetized mice (*n* = 5–6 mice per group). Microdialysis experiments were conducted in freely moving mice 24 and 48 h after surgery. Probes were perfused with artificial cerebrospinal fluid (aCSF in mM: NaCl, 125; KCl, 2.5; CaCl_2_, 1.26 and MgCl_2_ 1.18) at 1.5 μL/min. Following an initial 150 min stabilization period, five baseline samples were collected (20 min each) before local drug application by reverse dialysis and then successive dialysate were recovered. The concentration of glutamate and GABA in dialysate samples was determined by an HPLC system consisting of a Waters 717 plus autosampler, a Waters 600 quaternary gradient pump, and a Waters XBridge Shield RP18 5 μm 100 × 3 mm column. Dialysate samples were pre-column derivatized with OPA reagent by adding 90 μL distilled water to the 10 μL dialysate sample and followed by the addition of 15 μL of the OPA reagent. After 2.5 min reaction, 80 μL of this mixture was injected into the column. Detection was carried out with a Waters 2475 scanning fluorescence detector using excitation and emission wavelengths of 360 nm and 450 nm, respectively. The mobile phase was pumped at 0.8 μL/min and consisted of two components: solution A, made up of 0.05 M Na2HPO4, 28% methanol, adjusted to pH 6.4 with 85% H_3_PO_4_, and solution B, made up of 100% methanol/H_2_O (8:2 ratio). After the elution of glutamate peak at 2,10 min with 100% solution A, a gradient was established going from 100% solution A to 100% solution B in 2 min. After washing out eluting peaks (3 min), next phase returned to initial conditions (100% solution A) in 2 min, GABA elution were found at 11,6 min. The detection limit for glutamate and GABA was 0.2 pmol (signal-to-noise ratio 3). Quantification of Glu and GABA was carried out by comparison to a daily standard curve comprising the concentration of neurotransmitters expected in dialysate samples. Microdialysis data are expressed as femtomoles per fraction (uncorrected for recovery) and are shown in figures as percentages of basal values (individual means of 5 pre-drug fractions).

All reagents used were of analytical grade and were obtained from Merck (Germany). (S)-AMPA (AMPA, alpha-amino-3-hydroxy-5-methyl-4-is-oxazole-4-propionate) and NBQX (2,3-dihydroxy-6-nitro-7-sulfamoyl-ben-zo(f)quinoxaline) were from Sigma/RBI. Drugs were dissolved in the perfusion fluid. Concentrated solutions (1 mM; pH adjusted to 6.5–7 with NaHCO_3_ when necessary) were stored at – 80 °C, and working solutions were prepared daily by dilution in aCSF and administered by reverse dialysis at the stated concentrations.

### Statistical analysis

Statistical analyses were carried out using the GraphPad Prism 8.0 software. Sample sizes were chosen using a power analysis: 0.05 alpha value, 1 estimated sigma value and 75%. No methods of randomization were used to allocate animals to experimental groups. For single comparisons and normally distributed data (the Shapiro–Wilk normality test), we used two-tailed Student's *t* test (95% confidence). For multiple comparisons we used two-way ANOVA. For multiple comparisons, we used Kruskal–Wallis test plus Dunn’s multiple comparisons or Tukey’s test as a post hoc tests when required. A *p* value < 0.05 was considered significant. Non-normally distributed data were compared with Mann–Whitney *U* test for independent samples. All statistical information is further described in supplementary statistics.

## Results

### Cortical and thalamic synaptic inputs to the striatum are altered early in the R6/1 mouse model of Huntington’s Disease

**Fig. 1:**
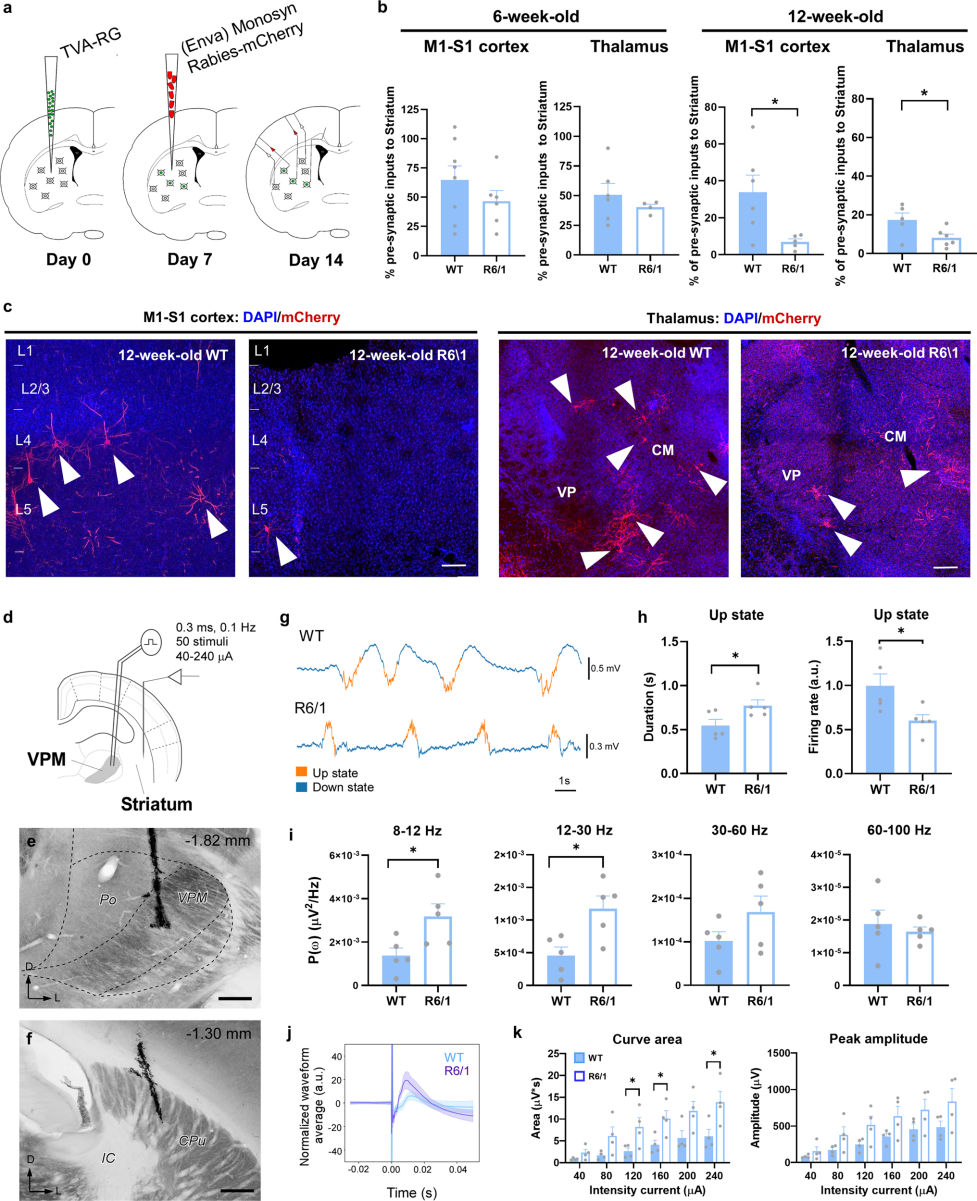
Striatal synaptic inputs assessment in the R6/1 mouse model. **a** Schematic representation of the experimental design for the Rabies-virus approach. **b** Histograms showing quantitative analysis of cortical and thalamic pre-synaptic inputs in 6- (left panels) or 12-week-old R6/1 (right panels) and WT mice. **c** Representative micrographs of pre-synaptic inputs in 12-week-old R6/1 and WT mice M1-S1 cortex (left panels) and thalamus (right panels). **d** Schematic representation of the experimental setup for in vivo electrophysiological recordings in a representative coronal section in 12-week-old WT and R6/1 mice. **e**, **f** Representative image of a mouse brain section stained for cytochrome oxidase showing the location of the bipolar electrode tip in the VPM (**e**) and the tungsten electrode in the striatum **f**. Scale bars: 250 µm. The anteroposterior level respect to bregma is indicated (upper right corner). **g** Spontaneous local field potentials (LFP) were recorded during slow oscillation activity in deeply anesthetized mice. Representative traces are shown. Up and down events are in orange and blue respectively. **h** Quantification of the Up states mean duration (in seconds; left panel) and the mean firing rate (arbitrary units; right panel) during the Up states. **i** Averaged power spectral density (PSD) over the z-scored normalized LFP of oscillatory activity at different frequency bands (alpha, 8–12 Hz; beta, 12–30 Hz; low-gamma, 30–60 Hz; high-gamma, 60–100 Hz). **j** Representative normalized evoked responses in the striatum after thalamic stimulation at 80 µA in WT (blue) and R6/1 (purple) mice. Solid lines, averaged waveforms; shaded areas, standard deviation. **k** The area under the curve (left panel) and the peak amplitude (right panel) were quantified for each current intensity tested, data in the range of 40–240 µA intensity currents are shown. Data are means ± SEM. * p < 0.05 compared to WT controls. Two-tailed unpaired t-test was employed in **b**, **f**, **i**, and **k**. n = 4–8 per genotype. *Po* posterior thalamic nucleus, *CPu* caudate putamen/striatum, *IC* internal capsule, *CM* Centro-medial nucleus, *VPM* ventral posteromedial nucleus

We first aimed to assess striatal network connectivity in the R6/1 mouse model of HD. To do so we used a monosynaptic circuit tracing system based on pseudotyped rabies virus [53] (Fig. 1a) in 6-week-old R6/1 mice (asymptomatic stage of the disease) and in 12-week-old R6/1 mice (early symptomatic stage of the disease) and age-matched wild type (WT) controls. This approach allowed us to perform a whole brain mapping of the areas that directly target the dorsal striatum such as motor and somatosensory cortices and the thalamus. We directly targeted the dorsal striatum because previous studies already described all pertinent leakiness controls for this method [54] (Supplementary Fig. 1a, b). First, at 6 weeks of age, R6/1 mice did not show changes of presynaptic inputs in cortex (S1-M1) or in thalamus (Fig. 1b) compared to age-matched WT controls. In contrast, at 12-week old, R6/1 mice showed a significant reduction in the proportion of presynaptic inputs from cortex and thalamus to the striatum (Fig. 1b, c) compared to WT controls. Although the cortico-striatal disconnection has already been widely described [4], the potential disconnection or aberrant connectivity between the thalamus and the striatum has poorly been addressed.

To address this point, we first recorded the spontaneous activity in the striatum of deeply anesthetized 12-week-old WT and R6/1 mice (Fig. 1d–f). In this state of deep anesthesia, the brain tends to generate a stereotypical pattern of slow oscillations consisting of alternating active (Up states) and silent (Down states) periods of neural activity [48, 55] (Fig. 1g). This activity pattern integrates cellular and connectivity properties of the network, and its quantification thus provides rich information about the underlying circuits in both physiological and pathological conditions [56–58]. We analyzed different parameters to characterize the striatal spontaneous activity. First, we found that the duration of the Up states in the striatum was longer in R6/1 than in WT mice, albeit with a decreased firing rate (Fig. 1g, h). Interestingly, R6/1 animals present higher spectral power values in the alpha (8–12 Hz), beta (12–30 Hz) and, partially, in the gamma (30–60 Hz) bands (Fig. 1i). We then investigated the evoked activity. To this end, we electrically stimulated the ventral posteromedial thalamic (VPM) nuclei and recorded the evoked responses in the striatum, triggered by intensities ranging from 40 to 240 µA. We observed that thalamo-striatal responses were larger in R6/1 compared to WT mice for the same stimulation intensity, showing a larger area under the curve and peak amplitudes for most of the range of intensities (Fig. 1j, k). These observations suggest an increase in the excitability or responsiveness of the striatum. We cannot rule out that it is secondary to an increased excitability of the presynaptic, thalamic nuclei. Previous intracellular studies in vitro have reported increased excitability of thalamic neurons in R6 lines with respect to WT mice at 6-–weeks, although these same neurons showed decreased thalamo-cortical transmission attributed to impaired neurotransmitter release [21]. Our results show that there are fewer thalamic neurons connecting with the striatum (Fig. 1a–c), deeming unlikely the thalamic origin of the hyperexcitability. Indeed, an increased responsiveness of the striatal neurons could be secondary to a homeostatic response to the decrease in thalamic and cortical synaptic inputs (review in [59]).

### Foxp2 levels are decreased in the thalamus of different mouse models of Huntington’s Disease

**Fig. 2:**
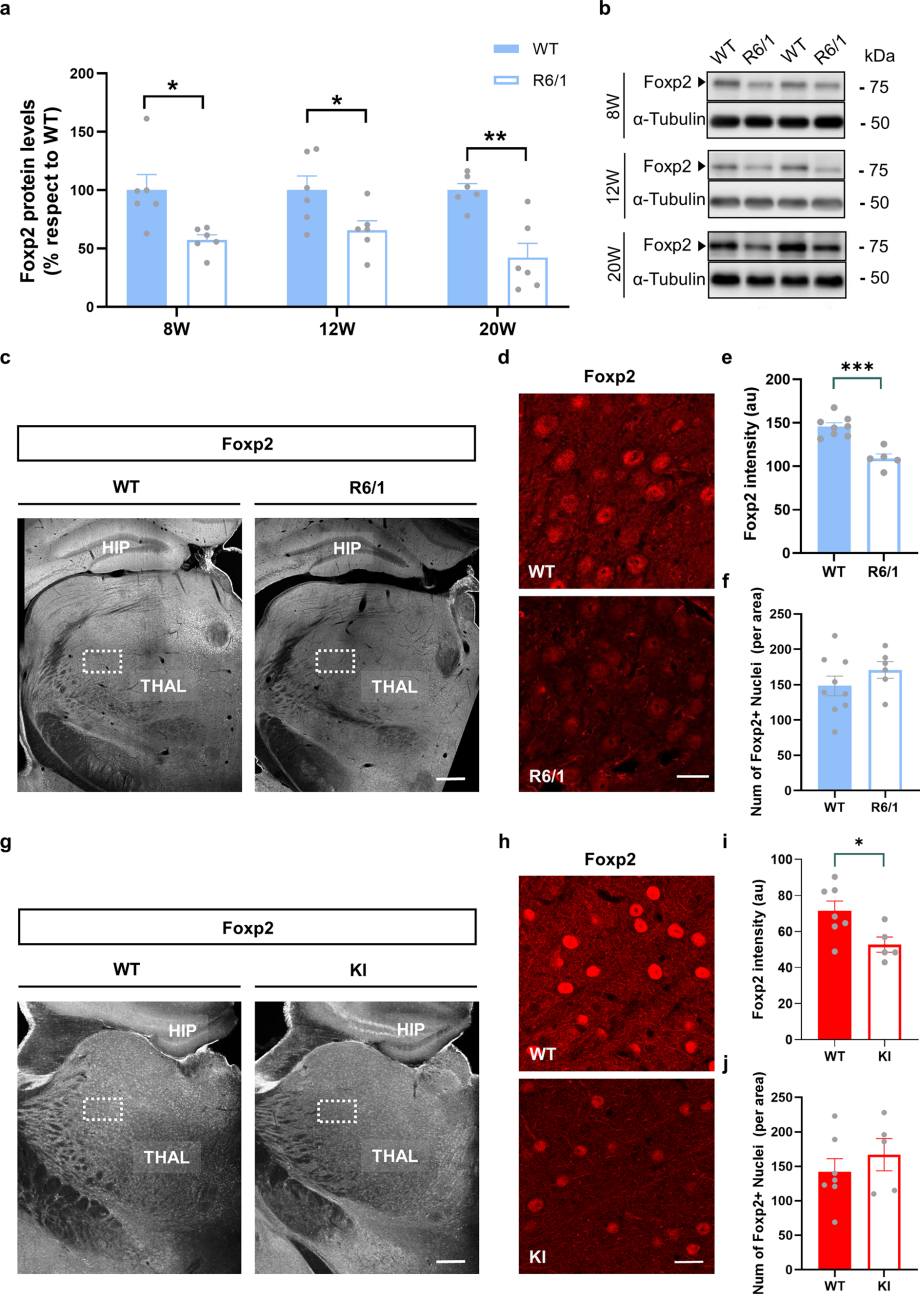
Thalamic Foxp2 protein levels in the R6/1 and Knock in mouse models of Huntington’s Disease. **a** Densitometric quantification of thalamic Foxp2 levels in WT and R6/1 mice at 8, 12 and 20 weeks of age. **b** Immunoblotting for Foxp2 and tubulin as a loading control. Data were normalized to tubulin. **c** Photomicrographs showing Foxp2 expression in coronal brain sections of 12-week-old WT (left panel) and R6/1 (right panel) mice. Scale bar, 500 µm. **d** Representative photomicrographs (high magnification) showing Foxp2 nuclei expression in the thalamus of WT controls (Upper panel) and R6/1 mice (Lower panels). Scale bar, 10 µm. We analyzed 1 image per slice and 3 slices per mouse. **e** Quantification of the intensity of integrated density (IOD, arbitrary units) and **f** number of Foxp2-positive nuclei/field in the thalamus. **g** Photomicrograph showing Foxp2 expression in coronal brain sections of 7-month-old WT (left panel) and knock-in (KI, right panel) mice. Scale bar, 500 µm. **h** Representative photomicrographs (high magnification) showing Foxp2 nuclei expression in the thalamus of WT (Upper panel) and KI mice (Lower panel). Scale bar, 10 µm. We analyzed 1 image per slice and 3 slices per mouse. **i** Quantification of the intensity of integrated density (IOD, arbitrary units) and **j** number of Foxp2-positive nuclei/field in the thalamus. Data are means ± SEM. Two-tailed unpaired t-test was employed in **a**, **e**, **f**, **i** and **j**. *p < 0.05, **p < 0.01 and ***p < 0.001 compared to WT controls. In **a** *n* = 6 mice/genotype, in **e–f** *n* = 5–8 mice/genotype, in **i**, **j** *n* = 5–7 mice/genotype

Since we observed an early aberrant thalamo-striatal connectivity and we previously described that Foxp2 could play a role in the early striatal synaptic function [26], we then hypothesized that Foxp2 could also play a role in this thalamo-striatal aberrant connectivity. To examine this, Foxp2 protein levels were analyzed in thalamic extracts obtained from R6/1 mice and WT controls. Thalamic samples were analyzed at different symptomatic stages: asymptomatic stage (8 weeks), early symptomatic stage (12 weeks), and late symptomatic stage (20 weeks). Remarkably, western blot analysis revealed that Foxp2 expression was significantly reduced at 8 weeks, 12 weeks, and 20 weeks in the transgenic R6/1 mice thalamic tissue compared to that in WT mice (Fig. 2a, b). To further investigate if the reduction of Foxp2 levels was restricted to different thalamic nuclei or, in contrast, was homogenous in the whole thalamus, we next performed an immunofluorescence against Foxp2 in 16-week-old R6/1 mice, when they show clear motor deficits [60], and WT age-matched controls. Foxp2 intensity was measured in coronal sections of the thalamus (Fig. 2c). We found a significant homogeneous reduction of Foxp2 protein levels in R6/1 thalamus compared to WT littermates (Fig. 2d, e) without altering the number of Foxp2-positive cells (Fig. 2d and f). To further investigate this reduction in the context of HD we then used another mouse model of the disease, the HdhQ111 knock-in (KI) mouse model at 7 months of age, when they show clear motor deficits [61]. As we did it before, Foxp2 intensity was measured in coronal sections of the thalamus (Fig. 2g). Once again, we found a significant homogeneous reduction of Foxp2 protein levels in KI thalamus compared to WT littermates (Fig. 2h, i) without altering the number of Foxp2-positive cells (Fig. 2h and j). Altogether these results point out to a broad reduction of thalamic Foxp2 levels in different mouse models of HD. Also, we hypothesize that, as a potential consequence, such reductions could play a role in the development of aberrant thalamo-striatal connections in HD.

### Foxp2 overexpression in the ventrobasal thalamus recovers sensorial and motor deficits in R6/1 mice

**Fig. 3:**
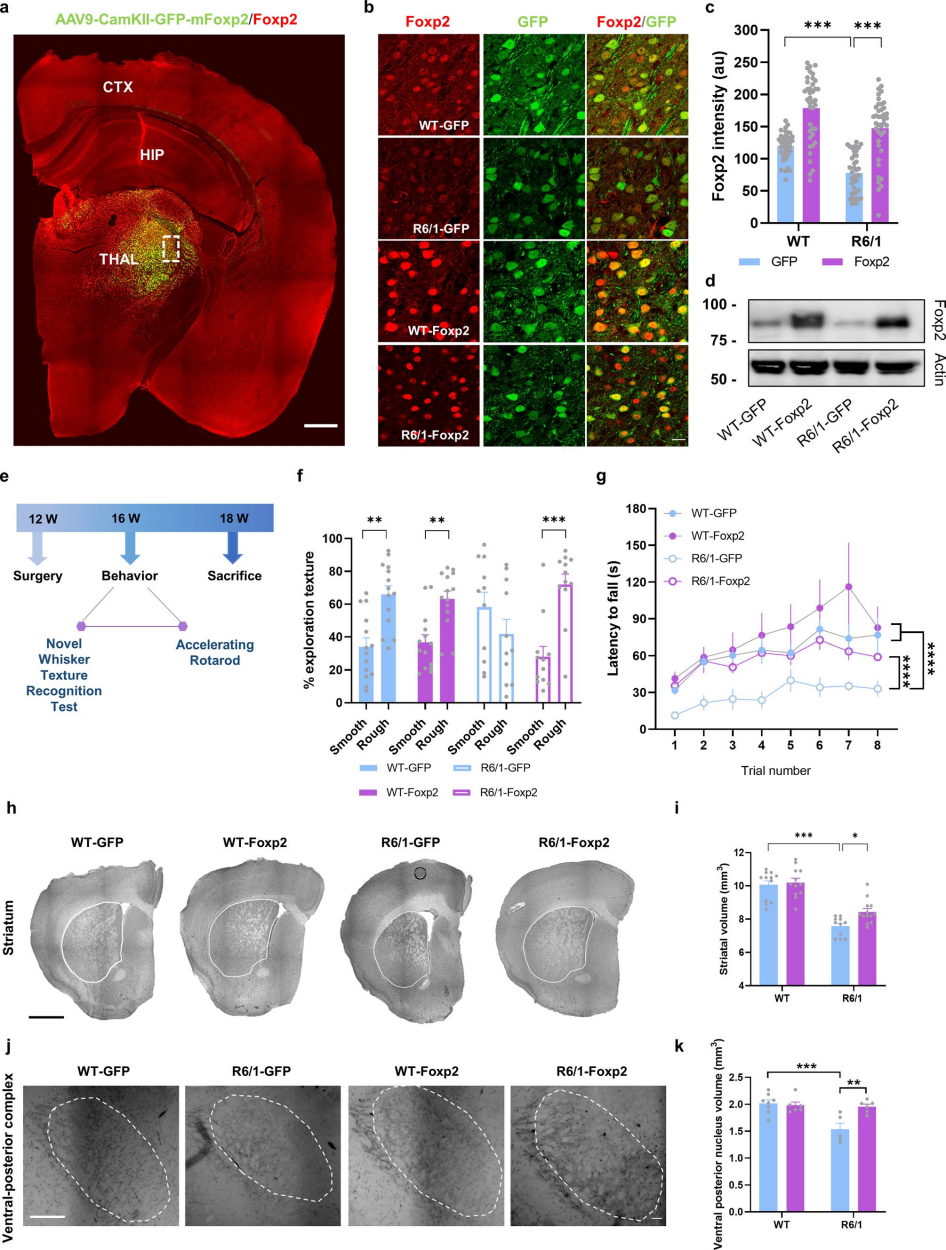
Restoration of thalamic Foxp2 levels and behavioral assessment of sensory-motor disturbances in the R6/1 mice. **a** Expression and distribution of Foxp2 (in red) in the transduced (in green) R6/1 mice. Scale bar, 500 µm. **b** Representative photomicrographs illustrating high magnifications of inset from **a** showing Foxp2 (red) nuclei expression and localization with GFP (green) in WT-GFP, R6/1-GFP (mice injected with a control AAV-CaMKII-GFP vector), WT-Foxp2 and R6/1-Foxp2 (mice injected with an AAV-CaMKII-Foxp2-GFP vector to over-express Foxp2) mice. Scale bar, 10 µm. **c** Quantification of the integrated optical density (IOD, arbitrary units) of the transduced Foxp2-positive nuclei in the thalamus as in **b**. Two-way ANOVA, group effect: *F*_(1,155)_ = 32.75; *p* < 0.0001; treatment effect: *F*_(1,38)_ = 100.3; *p* < 0.0001. (*n* = 40 nuclei per group, from 4 mice per group). **d** Results in c were verified by western blot. **e** Timeline represented in weeks (W) of behavioral testing in the transduced WT-GFP, R6/1-GFP, WT-Foxp2 and R6/1-Foxp2 mice. **f** % Time investigating texture object in the Novel Whisker Texture Recognition Test. Two-way ANOVA, object effect: *F*_(1,94)_ = 23.24; *p* < 0.0001; interaction effect: *F*_(3,94)_ = 7.940; *p* < 0.0001. **g** Latency to fall in the accelerating rotarod task. Repeated measures ANOVA, group effect: *F*_(3,26)_ = 4.039; *p* = 0.0175; time effect: *F*_(2.461, 63.96)_ = 13.78; *p* = 0.0006. **h** Representative photomicrographs of coronal brain sections (Nissl staining) from 18-week-old WT-GFP, R6/1-GFP, WT-Foxp2 and R6/1-Foxp2 mice. Scale bar, 1000 µm. **i** Striatal volume in all experimental groups was measured. Two-way ANOVA, group effect: *F*_(1,44)_ = 89.89; *p* < 0.0001. R6/1-GFP (*n* = 11); WT-GFP (*n* = 12), R6/1-Foxp2 (*n* = 13); WT-Foxp2 (*n* = 12). **j** Representative ventrobasal complex stained for cytochrome oxidase in 18-week-old WT-GFP, R6/1-GFP, WT-Foxp2 and R6/1-Foxp2 and mice. Scale bar, 200 µm. **k** Ventral posterior nucleus volume in all four groups was measured. Two-way ANOVA, group effect: *F*_(1,21)_ = 9.812; *p* = 0.0050 and interaction effect: *F*_(1,21)_ = 7.125; *p* = 0.0144. Data are means ± SEM. In **f**, **g**, WT-GFP (*n* = 14), R6/1-GFP (*n* = 11), WT-Foxp2 (*n* = 14) and R6/1-Foxp2 (*n* = 12). In **h–k**, WT-GFP (*n* = 8), R6/1-GFP (*n* = 5), WT-Foxp2 (*n* = 6) and R6/1-Foxp2 (*n* = 6). Tukey's post hoc test was used, **p < 0.01, ***p < 0.001 and ****p < 0.0001

To address whether the reduced Foxp2 levels are involved with the motor and sensory deficits previously described in the R6/1 mouse model [62, 63], we decided to rescue the levels of Foxp2 in the ventrobasal thalamic complex because it projects to the dorsolateral striatum [64], which is involved in motor coordination and learning consolidation [65, 66]. In addition, the VPM, which includes the ventrobasal complex, targets the layer IV of the primary somatosensory cortex (barrel cortex), which processes neuronal information of whisker-sensory and tactile perception in mice [67]. Therefore, we transduced the ventrobasal thalamus of 12-week-old R6/1 mice and WT mice (WT-Foxp2 and R6/1-Foxp2 groups) with an AAV to express Foxp2 to recover its loss in thalamic neurons of R6/1 mice (Fig. 3a–d). As a control we used WT and R6/1 mice injected in the same location with the same AAV but expressing only GFP (WT-GFP and R6/1-GFP groups). Three weeks after surgery, WT-GFP, WT-Foxp2, R6/1-GFP, and R6/1-Foxp2 mice were subjected to the novel whisker-dependent texture discrimination test and the accelerated rotarod test (Fig. 3e). First, we verified the effects of the local injection of the AAVs on normalization of Foxp2 protein levels in the injection site by immunofluorescence (Fig. 3b, c) and western blot (Fig. 3d). Our results showed a clear increase and total rescue of Foxp2 immunoreactivity in the thalamus of the R6/1-Foxp2 compared with WT-GFP mice (Fig. 3c). Next, we assessed whisker-dependent sensory-discrimination skills (Fig. 3e, f) and found that R6/1-GFP mice showed sensory deficits by spending similar times exploring the familiar smoot texture and the novel rough texture. In contrast, R6/1-Foxp2 mice displayed an improved sensory-whisker discrimination, reaching a similar performance to that displayed by WT-GFP mice (Fig. 3f). Then, to evaluate whether restoration of Foxp2 levels in the ventrobasal thalamus could also alleviate motor deficits, all experimental groups were subjected to an accelerated rotarod test, and latency to fall was measured. Latency to fall from the rod was decreased in R6/1-GFP mice compared with WT-GFP mice whereas the same parameter was completely rescued in R6/1-Foxp2 mice which reached similar levels of performance compared with WT-GFP mice (Fig. 3g).

We then sought whether those behavioral improvements could be related with potential improvements in neuroanatomical parameters of the cortico-thalamus-striatum loop [68]. We found that the striatal volume of R6/1-GFP mice was significantly reduced compared with WT-GFP mice and that this striatal atrophy was significantly improved in R6/1-Foxp2 mice (Fig. 3h, i). This result suggests that Foxp2 overexpression slightly delayed striatal neurodegeneration in R6/1 mice. Additionally, since Foxp2 is a regulator of thalamic identities and projection patterns [33], we performed a cytochrome oxidase staining and then analyzed the volume of ventral posterior thalamic complex in all four groups of mice. We observed that the volume of the ventral posterior thalamic complex of R6/1-GFP mice was reduced compared with WT mice but completely preserved in R6/1-Foxp2 mice (Fig. 3j, k). Altogether, these data suggest that overexpression of Foxp2 levels in thalamic neurons significantly recovered whisker dependent sensory discrimination, motor learning and associated gross neuroanatomical deficits observed in R6/1 mice.

### Foxp2 overexpression in the ventrobasal thalamus prevents cellular atrophy and synaptic loss in R6/1 mice

We then aimed to elucidate whether Foxp2 overexpression in thalamic neurons also correlated with changes in dorsal striatal neural subpopulations and structural synaptic plasticity of the cortico-thalamus-striatum loop. First, we evaluated the number and morphology of medium spiny neurons (MSNs) in all four groups using the DARPP-32 marker. The number of MSNs (Supplementary Fig. 2a, b), their DARPP-32 protein levels (Supplementary Fig. 2a and c) and their size (Supplementary Fig. 2a and d) were all decreased in R6/1-GFP mice but unaffected in R6/1-Foxp2 mice. We also assessed the state of other types of striatal resident interneurons such as parvalbumin- (PV) and ChAT-positive neurons but, although we found a general genotype effect, no changes were induced by Foxp2 over-expression (Supplementary Fig. 2e–h).

**Fig. 4:**
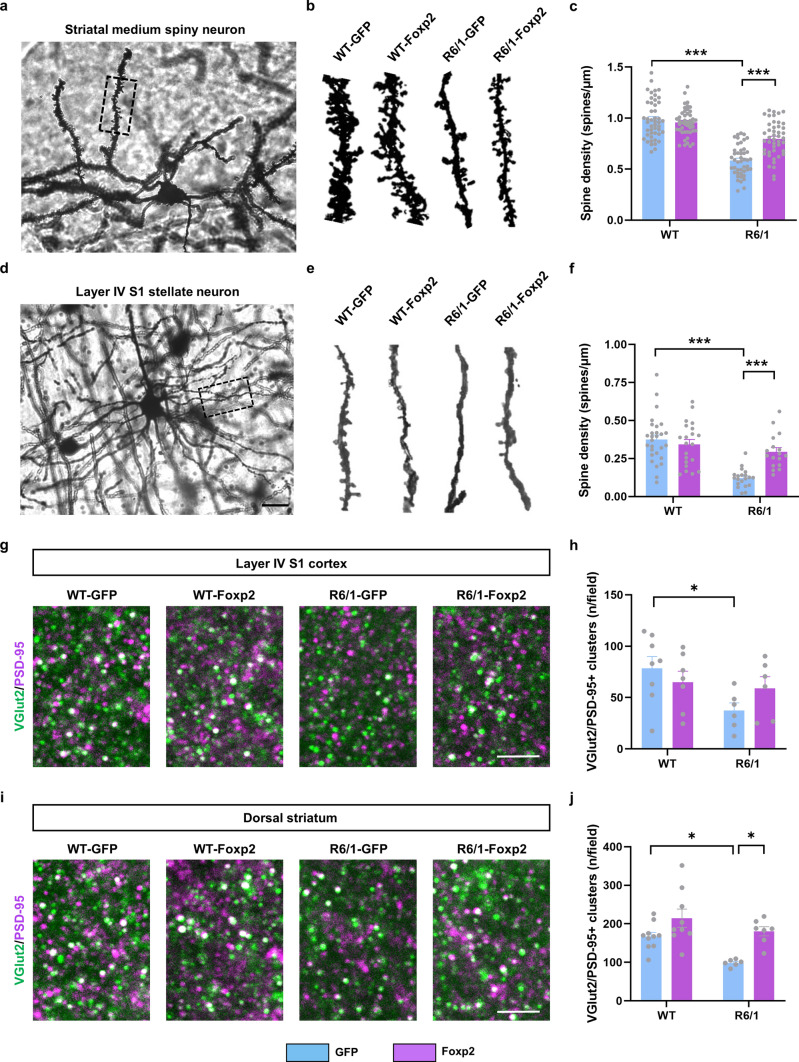
Structural synaptic plasticity characterization of WT and R6/1 mice transduced with AAV-Foxp2. **a** Representative photomicrograph showing a Golgi-cox-stained medium spiny neuron (MSN) and **b** representative Golgi-cox-stained dendrites in MSNs from 18-week-old WT-GFP, R6/1-GFP, WT-Foxp2 and R6/1-Foxp2 mice. **c** Graph showing quantitative analysis of dendritic spine density in all four groups. Two-way ANOVA, group effect: *F*_(1,179)_ = 136.8; *p* < 0.0001 and interaction effect: *F*_(1,179)_ = 25.11; *p* < 0.0001. (*n* = 4 animals per group, 10–12 dendrites per animal). **d** Representative photomicrograph showing a Golgi-cox-stained stellate neuron from layer IV of the S1 cortex (Scale bar 15 µm) and **e** representative Golgi-cox-stained dendrites of stellate neurons from WT-GFP, R6/1-GFP, WT-Foxp2 and R6/1-Foxp2 mice. **f** Graph showing quantitative analysis of dendritic spine density in all four groups. Two-way ANOVA, group effect: *F*_(1,82)_ = 29.61; *p* < 0.0001; and interaction effect: *F*_(1,82)_ = 13.49; *p* = 0.0004. (*n* = 4–5 animals per group, 7–10 dendrites per animal). **g** Confocal image of a double PSD-95 (magenta) and VGlut2 (green) immunofluorescence in layer IV of the S1 cortex of 18-week-old WT-GFP, R6/1-GFP, WT-Foxp2 and R6/1-Foxp2 mice. Scale bar, 6 μm. **h** Quantification of the number of PSD-95/VGlut2-positive puncta (white) per field. Two-way ANOVA genotype effect, *F*_(1,23)_ = 4.801, *p* = 0.0388. WT-GFP (*n* = 8), R6/1-GFP (*n* = 6), WT-Foxp2 (*n* = 7) and R6/1-Foxp2 (*n* = 6). **i** Confocal image of a double PSD-95 (magenta) and VGlut2 (green) immunofluorescence in dorsal striatum of 18-week-old WT-GFP, R6/1-GFP, WT-Foxp2 and R6/1-Foxp2 mice. Scale bar, 6 μm. **j** Quantification of the number of PSD-95/VGlut2-positive puncta (white) per field. Two-way ANOVA genotype effect, *F*_(1,28)_ = 9.840, *p* = 0.0040; treatment effect, *F*_(1,28)_ = 15.72, *p* = 0.0005. WT-GFP (*n* = 10), R6/1-GFP (*n* = 6), WT-Foxp2 (*n* = 9) and R6/1-Foxp2 (*n* = 7). In **g**–**j** we analyzed 2 images per slice and 2 slices per mouse. Data are means ± SEM in all experiments. Tukey's post hoc test was used *p < 0.05, **p < 0.01, ***p < 0.001 and ****p < 0.0001

Then, we analyzed dendritic spine density in Golgi-impregnated dorsal MSNs. As previously described [69], we also observed that dendritic spine density was reduced in dorsal MSNs from R6/1-GFP mice compared to WT-GFP mice. However, Foxp2 overexpression in the ventrobasal thalamus increased the spine density in the dorsal MSNs of R6/1-Foxp2 mice compared with those in R6/1-GFP mice (Fig. 4a–c). Finally, we also analyzed the dendritic spine density in Golgi-impregnated stellate cortical neurons (located in the layer IV of the barrel cortex (S1) in the same four groups of mice. The dendritic spine density in stellate cortical neurons from R6/1-GFP mice was reduced compared to WT-GFP (Fig. 4d–f). In contrast, R6/1-Foxp2 mice had a significant recovery of spine density compared with R6/1-GFP mice (Fig. 4d–f). Deepen on dendritic spine pathology, we also evaluated spine length and spine head diameter in both neuronal types. First, spine head diameter and spine length were both reduced in R6/1-GFP MSNs (Supplementary Fig. 3a, b) and stellate neurons (Supplementary Fig. 3c, d). Interestingly, all these parameters were recovered in R6/1-Foxp2 except for spine length in stellate neurons that was even longer in R6/1-Foxp2 mice than in WT-GFP mice (Supplementary Fig. 3d).

Finally, we explored the structural thalamo-striatal and thalamo-cortical synaptic connectivity. To do so, we evaluated the colocalization of VGlut2-positive clusters (as a marker of pre-synaptic glutamatergic terminals from the thalamus) with PSD-95-positive clusters (glutamatergic post-synaptic marker) in dorsal striatum and in layer IV of the S1 cortex. We found that the number of double VGlut2/PSD-95 clusters was reduced in R6/1 mice but not affected in R6/1-Foxp2 mice compared with WT-GFP mice (Fig. 4g, h). Similar results were obtained when we moved to the dorsal striatum; VGlut2/PSD-95 clusters were dramatically reduced in R6/1-GFP mice but fully recovered in R6/1-Foxp2 mice (Fig. 4i, j). In summary, our results indicate that thalamic Foxp2 levels are crucial for proper dorsal MSNs states and for the preservation of thalamo-striatal and thalamo-cortical synapses in HD.

### Foxp2 overexpression in the ventrobasal thalamus normalizes spontaneous and evoked striatal and cortical activity in R6/1 mice

**Fig. 5:**
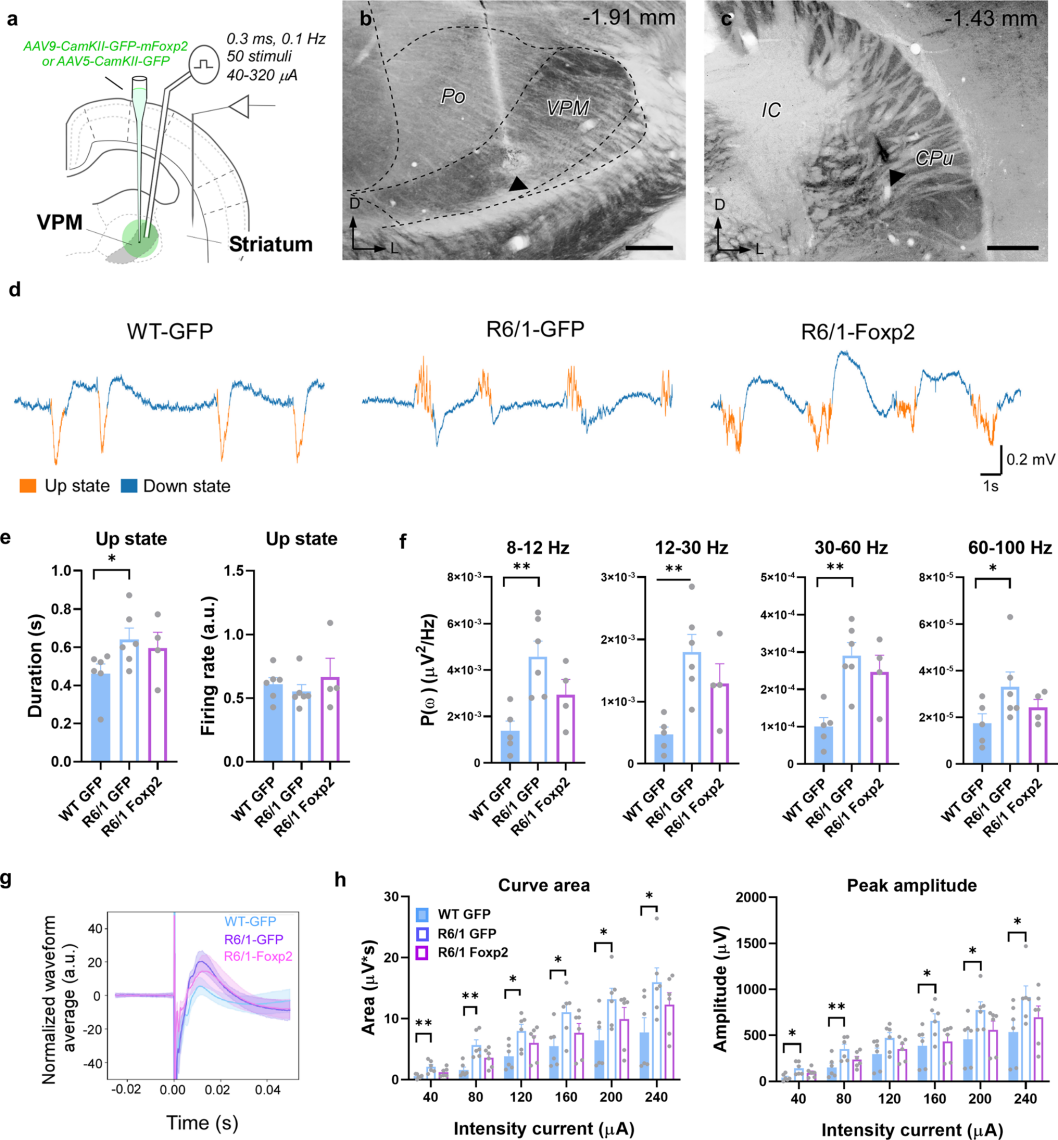
Effects on spontaneous activity and thalamo-striatal evoked responses upon Foxp2 levels recovery in the R6/1 mice thalamus. **a** Schematic representation of the experimental setup in a representative coronal section. Stereotaxic viral injections were performed bilaterally in the thalamus of 12-week-old mice to produce an overexpression of Foxp2. After four weeks of AAV expression, a bipolar stimulation electrode was placed in the thalamus to elicit striatal responses in the anesthetized 16-week-old mice. **b**, **c** Representative images of a coronal brain section counterstained with cytochrome oxidase showing the location of the bipolar electrode tip in VPM **b** and the tungsten electrode in the striatum **c** in a R6/1 mouse. Scale bars: 250 µm. The anteroposterior level respect to bregma is indicated in the upper right corner. Black arrowheads indicate the tip of the electrodes. **d** Spontaneous local field potentials (LFP) were recorded during slow oscillation activity in deeply anesthetized mice. Representative raw traces of the LFP in the three experimental groups (WT-GFP, R6/1-GFP and R6/1-Foxp2) are shown. Up and down events are colored in orange and blue, respectively. **e** Quantification of the Up states mean duration (in seconds; left panel) and the mean firing rate (arbitrary units; right panel) during the Up states. **f** Averaged power spectral density (PSD) over the z-scored normalized LFP of oscillatory activity at different frequency bands (alpha, 8–12 Hz; beta, 12–30 Hz; low-gamma, 30–60 Hz; high-gamma, 60–100 Hz) in WT-GFP, R6/1-GFP and R6/1-Foxp2 mice. **g** Representative normalized evoked responses in striatum after thalamic stimulation at 80 µA in WT-GFP (blue), R6/1-GFP (purple) and R6/1-Foxp2 (pink) mice. Solid lines indicate the trial-averaged waveforms while the shaded areas indicate the standard deviation from the mean. **h** The area under the curve (left panel) and the peak amplitude (right panel) were quantified for each current intensity tested, data in the range of 40–240 µA intensity currents are shown. Data are represented as mean ± SEM. Kruskal–Wallis test was used, *p < 0.05, **p < 0.01 compared with WT-GFP mice. *Po* posterior thalamic nucleus, *CPu* caudate putamen/striatum, *IC* internal capsule, *VPM* ventral posteromedial nucleus

We then deepened on whether structural and functional improvements observed in R6/1 mice after their thalamic Foxp2 restoration were associated with physiological changes in thalamo-striatal and thalamo-cortical connections. To do so we first recorded the spontaneous activity in the striatum of deeply anesthetized WT-GFP, R6/1-GFP and R6/1-Foxp2 mice (Fig. 5a–c). As it was observed in the sample of animals not expressing GFP (Fig. 1d–i), we found once again that Up states in the striatum were longer in R6/1-GFP than in WT-GFP mice, whereas in the R6/1-Foxp2 mice, Up-duration values were comprised between the WT-GFP and R6/1-GFP ones, without significant differences in comparison with the WT-GFP group (Fig. 5d, e) and suggesting a partial recovery of these parameters. Intriguingly, we did not detect changes on firing rate in R6/1-GFP mice (Fig. 5e, right panel) as we did it in naïve R6/1 mice (Fig. 1h, right panel). Regarding the spectral power values in the alpha (8–12 Hz), beta (12–30 Hz) and gamma (30–60 Hz) bands, we validated again the higher values in R6/1-GFP mice compared with WT-GFP (Fig. 5f). Interestingly, these parameters in the R6/1-Foxp2 mice were reduced and statistically undistinguishable when compared with the values of WT-GFP mice (Fig. 5f). We then investigated the thalamic evoked activity in the striatum of WT-GFP, R6/1-GFP and R6/1-Foxp2 mice elicited by electrical stimulation in the VPM (Fig. 5g). We observed that thalamo-striatal responses were also larger in R6/1-GFP compared to WT-GFP mice, whereas in R6/1-Foxp2 these responses were non-significantly different from those observed in WT-GFP mice (Fig. 5g, h).

**Fig. 6:**
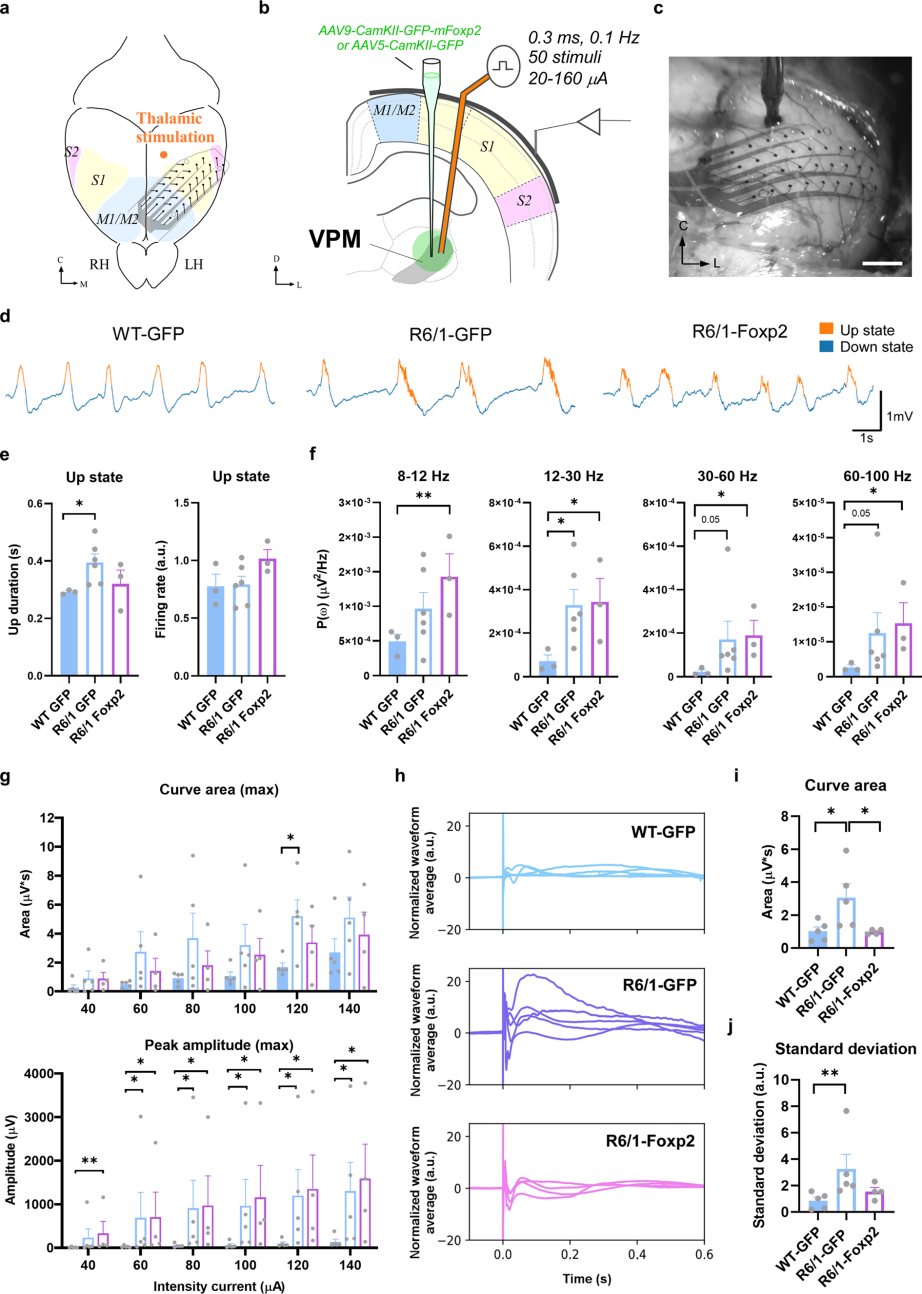
Effects on spontaneous activity and thalamo-cortical evoked responses upon Foxp2 levels recovery in the R6/1 mice thalamus. **a** Schematic representation of the experimental setup in a top view of the mouse brain and **b** a coronal section. The bipolar stimulation electrode was placed in VPM thalamic nucleus to elicit cortical responses in anesthetized mice. LFP were recorded from different motor cortex (M1/M2) and the primary (S1) and secondary (S2) somatosensory cortical areas through a superficial 32-channels MEA. **c** Microphotograph showing the array placed in the cortical surface of left hemisphere during an experiment. Scale bar: 500 µm. **d** Representative raw traces of the local field potentials (LFP) in the three experimental groups (16-week-old WT-GFP, R6/1-GFP and R6/1-Foxp2 groups of mice) are shown. Up and down events are colored in orange and blue, respectively. **e** Quantification of the Up states mean duration (in seconds; left panel) and the mean firing rate (arbitrary units; right panel) during the Up states. **f** Averaged power spectral density (PSD) over the z-scored normalized LFP of oscillatory activity at different frequency bands (alpha, 8–12 Hz; beta, 12–30 Hz; low-gamma, 30–60 Hz; high-gamma, 60–100 Hz) in WT-GFP, R6/1-GFP and R6/1-Foxp2 mice. **g** The evoked responses in the cortex in the first 50 ms after the stimulation. The responses evoked in the 32 channels were quantified, and the maximum area under the curve (upper panel) and maximum peak amplitude (lower panel) of the first wave are shown for the range of 40–140 µA intensity currents. **h** Long-lasting baseline z-score normalized evoked responses (in a.u.) after thalamic stimulation at 160 µA in WT-GFP (blue; upper panel), R6/1-GFP (purple; middle panel) and R6/1-Foxp2 (pink, lower panel) mice. Solid lines indicate the trial waveforms of each animal. **i** The area under the curve was quantified for a time-window of 600 ms post-stimuli using the 160 µA intensity current protocol for each experimental group. **j** The value distribution of the area under the curve of the normalized evoked responses in the 32 channels recorded was quantified as the standard deviation (in a.u.). Data are represented as mean ± SEM. Kruskal–Wallis test was used, *p < 0.05, **p < 0.01 compared with WT-GFP mice. *RH* right hemisphere, *LH* left hemisphere, *M1/M2* motor areas 1 and 2, *S1* sensory area, *VPM* ventral posteromedial nucleus, *C* center, *L* left

Since recovering Foxp2 levels in the thalamus of R6/1 mice exerted such rescue of striatal and thalamo-striatal electrophysiological parameters we next investigated whether similar electrophysiological findings, including recovery, could be detected in the cortical circuits and thalamo-cortical connections, given the relevance of cortical inputs on striatal internal dynamics [70]. As a first step, we recorded the spontaneous activity in the motor and somatosensory cortices of naïve WT and R6/1 mice under deep anesthesia using a 32 channels multi-electrode array (Fig. 6a–f). Specifically, measures of Up states were the result of the average for all the electrodes. However, the measures of the evoked responses corresponded to the electrodes in S1, main target of VPM. As for the striatum, here we first describe the cortical oscillatory activity of WT and R6/1 (Supplementary Fig. 4a–g). We observed that the duration of the Up states was also longer in the cortex of R6/1 than in WT animals (Supplementary Fig. 4b), as it was the case in the striatum (Figs. 1h and 5e). Similarly, Up states were also longer in the cortex of R6/1-GFP mice when compared with WT-GFP mice (Fig. 6d, e). Contrary, the recovery of thalamic Foxp2 levels in R6/1-Foxp2 mice produced a slight reduction in the duration of the Up states and no differences were found between R6/1-Foxp2 and WT-GFP groups (Fig. 6d, e). As described as well for the striatum (Fig. 5f), R6/1 animals also presented in the cortex higher spectral power values in the beta (12–30 Hz) and gamma (30–100 Hz) bands when compared to WT mice (Supplementary Fig. 4c). Likewise, the same increase in spectral power values was observed in R6/1-GFP mice in the cortex when compared with WT-GFP, although no recovery was observed in R6/1-Foxp2 compared with WT-GFP mice (Fig. 6f).

Next, we applied electrical stimulation in the VPM and recorded the evoked responses in the cortex, triggered by intensities ranging from 20 to 140 µA. As in the striatum, we also found an increase in the responsiveness in the cortex, being higher both the area and peak amplitudes in naïve R6/1 mice for both the early response (1st peak) and the late response (2nd peak) (Supplementary Fig. 4d–g). When comparing the R6/1-GFP with the WT-GFP mice, no significant differences were observed in the area of the response, although there was an increase in the amplitude of the response (Fig. 6g). Nevertheless, no recovery was observed in R6/1-Foxp2 when compared with either, WT-GFP or R6/1-GFP mice (Fig. 6g). Remarkably, when we analyzed the long-lasting evoked normalized responses in a wider time window (600 ms) following the stimulation, which better reflects the cortical network responsiveness and the inter-areal intercommunication than the early postsynaptic thalamo-cortical responses, we observed that the recovery of the Foxp2 levels in the thalamus produces smaller responses in R6/1 animals, being similar to the ones found in WT-GFP (Fig. 6h–j). This is suggestive that, even when the striatum is more responsive than the cerebral cortex to the restoration of Foxp2 levels, some aspects of cortical network dynamics that are affected in the R6/1 model can be partially recovered by increasing the levels of the Foxp2 in the thalamus, emphasizing the potential of this strategy.

In summary, the recovery of thalamic Foxp2 levels in R6/1 induced a partial rescue of the aberrant thalamo-striatal functional connectivity and a partial recovery of some of their impairments in cortical network dynamics.

### Foxp2 overexpression in the ventrobasal thalamus corrects in vivo striatal glutamate and GABA release in R6/1 mice

**Fig. 7:**
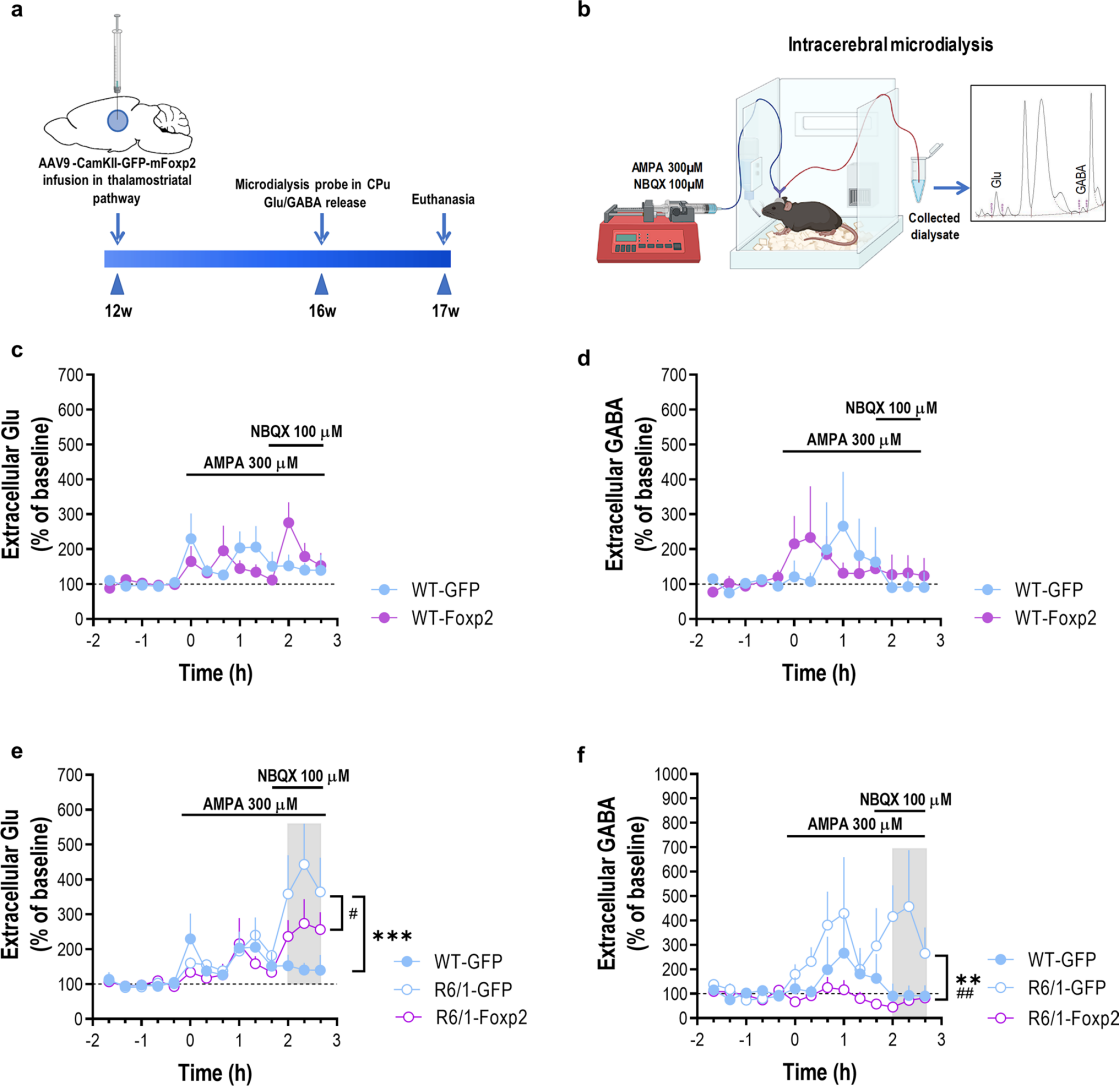
Expression of Foxp2 in the thalamus and its regulation of Glu/GABA neurotransmission in a R6/1 mouse model. **a** Timeline of microdialysis experimentation in the transduced WT-GFP, R6/1-GFP, WT-Foxp2 and R6/1-Foxp2 mice. **b** Schematic diagram showing an in vivo microdialysis procedure to measure Glu/GABA release. Mice were implanted with one dialysis probe in the striatum and after an initial stabilization period, five baseline samples were collected following by local drug application by reverse dialysis. The concentration of Glu and GABA in dialysate samples was determined by an HPLC system with fluorescence detection. The inset shows a typical chromatogram of Glu/GABA in mouse striatum. **c** AMPA (300 µM) induced a similar effect on Glu release in the striatum of WT-GFP (*n* = 6) and WT-Foxp2 (*n* = 5) mice. The co-perfusion of the AMPA receptor antagonist NBQX (100 µM) attenuated AMPA effect on Glu levels. Two-way ANOVA showed a time effect (*F*_13,126_ = 2.532; *p* = 0.0040). **d** In parallel, striatal application of AMPA + NBQX elicited a similar effect on GABA release in the same mouse groups. **e** Notably, co-perfusion of NBQX potentiated AMPA effect on Glu release in the striatum of R6/1-GFP mice (*n* = 6), but not in R6/1-Foxp2 mice (*n* = 5). Two-way ANOVA showed a genotype effect (*F*_2,196_ = 6.145; *p* = 0.0026), time (*F*_13,196_ = 6.773; *p* < 0.0001), and interaction genotype-by-time (*F*_26,196_ = 1.738; *p* = 0.0189). **f** The co-perfusion of NBQX was also able to increase striatal GABA release in R6/1-GFP mice, an effect prevented in R6/1-Foxp2 mice. Two-way ANOVA showed a genotype effect (*F*_2,182_ = 12.81; *p* < 0.0001). Data are as mean ± SEM. Two-way ANOVA and Bonferroni's multiple comparisons test, *p < 0.05, **p < 0.01 and ***p < 0.001 compared to WT-GFP mice; ^**#**^*p* < 0.05, ^**##**^*p* < 0.01 compared to R6/1-Foxp2 mice

We next performed microdialysis experiments (Fig. 7a, b) in the striatum of freely moving mice (four groups, WT-GFP, WT-Foxp2, R6/1-GFP, and R6/1-Foxp2) at 16 weeks to examine whether the Foxp2 overexpression in the ventrobasal thalamus affects glutamate (Glu) and GABA neurotransmission. No differences in baseline extracellular Glu and GABA concentration were found between the different groups (Supplementary Table 1). Next, local application of 300 µM AMPA (AMPA receptor agonist) by reverse dialysis slightly increased striatal Glu levels in both WT-GFP and WT-Foxp2 control groups. The co-perfusion of NBQX (100 µM, selective AMPA receptor antagonist) attenuated the Glu enhancing effect of AMPA (Fig. 7c). In parallel, both control mice (WT-GFP and WT-Foxp2) did not show significant changes in striatal GABA levels after AMPA + NBQX application (Fig. 7d). Since both control groups showed similar effects on Glu and GABA neurotransmission in the striatum, we performed the following microdialysis analyses using WT-GFP as the control group.

Thus, while local infusion of 300 µM AMPA also slightly increased Glu levels in the striatum of R6/1-GFP and R6/1-Foxp2 mice, co-perfusion of NBQX (100 µM) produced a marked increase in Glu release in R6/1-GFP mice, but not in R6/1-Foxp2 mice, compared to WT-GFP group (Fig. 7e). Simultaneously, striatal GABA release showed no significant differences after local application of AMPA in either experimental group, however, co-perfusion of NBQX significantly increased extracellular GABA levels only in the striatum of R6/1-GFP mice compared to WT-GFP and R6/1-Foxp2 mice (Fig. 7f). These results suggest that recovery of Foxp2 in the thalamus pathway significantly rescues some aspects of striatal Glu/GABA neurotransmission in the R6/1 mouse model.

### Downregulation of Foxp2 levels in ventrobasal thalamus in wild type mice induces motor and whisker-sensory deficits

**Fig. 8:**
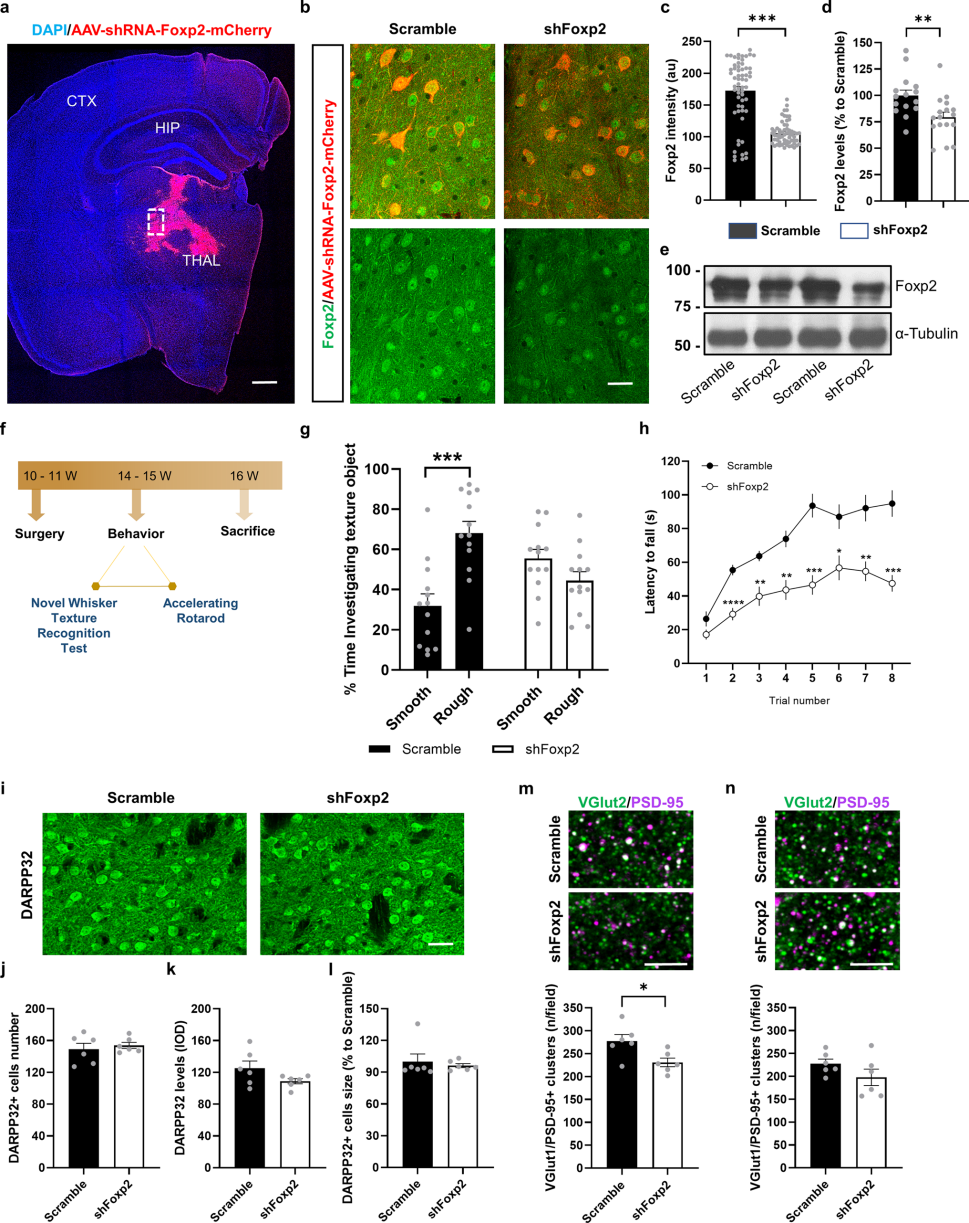
Behavioral assessment of sensory-motor disturbances in the WT-shFoxp2 mice. WT mice were injected in the thalamus with either, AAV-shRNAFoxp2-mCherry (shFoxp2 group) or AAV-Scramble-mCherry (Scramble group) viral constructs. **a** Distribution of transduced thalamus with AAV-shRNA-Foxp2-mCherry (in red) in a coronal brain section co-labeled with a DAPI staining (blue). Scale bar, 500 µm. **b** Representative photomicrographs illustrating high magnifications of inset from **a** showing Foxp2 (green) expression and localization in thalamic transduced neurons (in red). Scale bar, 25 µm. **c** Quantification of the Foxp2-positive integrated optical density (IOD, arbitrary units) in the nucleus of the transduced thalamic neurons as in **b**. Unpaired t-test; *p* < 0.0001. (*n* = 60 nuclei per group/10 nuclei per mouse/6 mouse per group). **d** Densitometric quantification of thalamic Foxp2 levels in Scramble and shFoxp2 mice. **e** Immunoblotting for Foxp2 and α-tubulin as a loading control. Data were normalized to tubulin. Unpaired *t* test; *p* < 0.006. **f** Timeline expressed in weeks (W) of behavioral testing in the Scramble and shFoxp2 groups of mice. **g** % Time investigating texture object in the Novel Whisker Texture Recognition Test was measured. Two-way ANOVA, object effect: *F*_(1,48)_ = 5.734; *p* = 0.0206 and interaction effect: *F*_(1,48_) = 20.3; *p* < 0.0001. Scramble group (*n* = 13) and shFoxp2 group (*n* = 13). Tukey's post hoc test was used ****p < 0.0001 to compare % time exploring the rough vs the smooth textures in each group. **h** Latency to fall in the accelerating rotarod task in Scramble and shFoxp2 groups of mice was measured. Repeated measures ANOVA, group effect: *F*_(1,31)_ = 28.16; *p* < 0.0001; and interaction effect: *F*_(7,217_) = 4.678; *p* < 0.0001. Scramble group (*n* = 13) and shFoxp2 group (*n* = 13). Bonferroni's post hoc test was used *p < 0.05, **p < 0.01, ***p < 0.001 and ****p < 0.0001 compared to Scramble mice. **i** Representative microphotographs of coronal sections from Scramble and shFoxp2 groups of mice immunostained for DARPP-32 in the dorsal striatum. Scale bar, 30 μm. **j** Number of DARPP-32-positive neurons/field. **k** DARPP-32 integrated optical density (IOD). **l** Soma size (µm^2^ relativized to the Scramble group) of DARPP-32-positive neurons. **m** Confocal images (upper panels) of a double PSD-95 (magenta) and VGlut2 (green) immunofluorescence in dorsal striatum of Scramble (upper image) and shFoxp2 (lower image) groups of mice. Scale bar, 6 μm. Quantification (lower graph) of the number of PSD-95/VGlut2-positive puncta (white) per field. Unpaired *t* test; *p* = 0.021. **n** Confocal image of a double PSD-95 (magenta) and VGlut2 (green) immunofluorescence in layer IV of the S1 cortex of Scramble (upper image) and shFoxp2 (lower image) groups of mice. Scale bar, 6 μm. Quantification (lower graph) of the number of PSD-95/VGlut2-positive puncta (white) per field. In **i**–**n** we analyzed 2 images per slice and 2 slices per mouse (Scramble (*n* = 6) and shFoxp2 (*n* = 6)). Data are means ± SEM in all experiments

To test whether lowering of Foxp2 levels in thalamic neurons of WT mice could mimic the HD motor-sensory disturbances, we transduced the ventrobasal thalamus of 11-week-old WT mice with an AAV expressing a shRNAFoxp2 (shFoxp2 group) or with an AAV expressing a scramble construct (Scramble group) (Fig. 8a–e). Three weeks after surgery, the groups of mice, Scramble and shFoxp2 groups, were subjected to the novel whisker-dependent texture discrimination paradigm and the rotarod test (Fig. 8f). We verified the injection sites (Fig. 8a), and the effects of Foxp2 gene knockdown in the injection site by immunofluorescence (Fig. 8b, c). Our results showed a clear decrease of Foxp2 immunoreactivity in the neurons of the ventrobasal thalamus (Fig. 8b, c). We confirmed these results by western blot (Fig. 8d, e). Next, we evaluated the whisker-dependent sensory discrimination (Fig. 8g). We found that shFoxp2 mice displayed an impairment in sensory-whisker discrimination compared to the Scramble group, suggesting that by only downregulating Foxp2 in the ventrobasal thalamus was enough to replicate HD-like whisker-sensory disturbances. Next, we also assessed motor learning and coordination in the accelerating rotarod test (Fig. 8h). Latency to fall from the rod was significantly decreased in the shFoxp2 group with respect to the Scramble group.

We then evaluated potential neuropathological changes in the dorsal striatum and in the layer IV of the cortical S1 from shFoxp2 and Scramble mice. First, we evaluated the number and morphology of MSNs using the DARPP-32 marker. The number of MSNs (Fig. 8i, j), the DARPP-32 protein levels (Fig. 8i and k) and size of MSNs (Fig. 8i and l) were all of them undistinguishable between Scramble and shFoxp2 groups. Intriguingly, we observed no changes in PV interneurons occupied area but a significant alteration in ChAT interneurons in shFoxp2 mice (Supplementary Fig. 5a–d). We then explored the structural thalamo-striatal and thalamo-cortical synaptic connectivity. We evaluated the colocalization of VGlut2-positive clusters with PSD-95-positive clusters in dorsal striatum, and in layer IV of the S1 cortex. In the dorsal striatum we found that the number of double VGlut2/PSD-95 clusters was reduced in shFoxp2 mice compared with Scramble mice (Fig. 8m). In contrast, double VGlut2/PSD-95 clusters in layer IV of the S1 cortex were not affected in shFoxp2 mice compared with Scramble mice (Fig. 8n). In summary, our results show that downregulating Foxp2 in the thalamus is enough to mimic some sensory and motor as well as synaptic HD-like phenotypes.

## Discussion

Using a monosynaptic circuit tracing system and in vivo electrophysiological recordings, in the present study we show an early thalamo-striatal aberrant connectivity at the same age when cortico-striatal disconnection takes place in the R6/1 mouse model of HD. We noticed that this altered thalamo-striatal connectivity was concomitant with an early, prominent, and persistent downregulation of thalamic Foxp2 protein levels. Such alteration on Foxp2 levels was observed in both, exon-1 and knock-in mouse models of HD. Genetic recovery of Foxp2 levels in the ventrobasal thalamus was accompanied by a recuperation in impaired thalamo-striatal and thalamo-cortical spontaneous and elicited electrophysiological activity as well as a rescue in sensorial and motor disturbances largely described in the R6/1 mouse model. In the same set of experiments, dendritic spine loss in stellate neurons of the layer IV of the sensorial cortex as well as in MSNs were both significantly recovered in R6/1 mice with normalized thalamic Foxp2 levels compared with R6/1 control mice. Moreover, the later result was associated with an amelioration of striatal and thalamic atrophies as well as with an improvement of the perturbed release of striatal GABA and glutamate. Finally, we were capable to mimic R6/1 sensorial and motor deficits by genetic inhibition of Foxp2 in neurons of the ventrobasal thalamus in WT mice.

As far as we know, the link of Foxp2 to the pathogenesis of HD was firstly proposed as a relevant pathway in 2017 by Oswald et al. [71]. They postulated that Foxp2 is connected with language and communication deficiencies as well as to neurotransmitters deficits, calcium dysfunction, and neuronal aberrant wiring [71]. In the present work we explored the aberrant thalamo-striatal and thalamo-cortical wiring. Our gain-of-function as well as loss-of-function experiments demonstrate that Foxp2 levels in thalamic neurons are essential in the modulation of the largely described motor and sensory impairments in the R6/1 mouse model of HD [60, 63]. Accordingly, thalamo-striatal afferents contribute to the acquisition of sequential motor learning by targeting the dorsal striatum [65]. Furthermore, loss of thalamo-striatal synaptic activity is associated with motor coordination impairments [72]. Additionally, the sensory impairments, already described in patients with HD [73], could also involve the thalamo-cortical connections [74, 75] implicating the stellate neurons of layer IV of the sensory cortex [76]. The latter disturbances have largely been related to alterations in micro-structural synaptic plasticity changes such as dendritic spine loss in MSNs as well as in stellate neurons [69, 77, 78] together with changes on their morphology [36, 79–82]. Such deficits in dendritic spine pathology were strongly recovered by rescuing thalamic Foxp2 expression. Altogether reinforce the idea that reduced thalamic Foxp2 levels play a crucial role in the development of motor and sensorial deficits and associated histopathological hallmarks in HD.

Our results also indicate that regional functionality in the ventrobasal thalamus is compromised in HD because of a reduction of Foxp2 levels in the resident thalamic neurons and that this reduction has a substantial impact on its outputs: to the striatum and to the primary somatosensory cortex in HD. As a functional underlying mechanism, we observed that GABA (and to a lesser extent, glutamate) release dynamics in the striatum were corrected due to a rescue of thalamic Foxp2 expression. In this sense, upregulated GABA release could also be a relevant process since all innervated striatal neurons by glutamatergic thalamo-striatal axons are GABAergic [83]. The origin of this increased intrastriatal GABAergic release in R6/1 mice is intriguing. For example, it has been described in HD models that, in the globus pallidus, GABA from the striatum is reduced [84, 85]. However, intra-striatal increased GABAergic activity has already been described in HD models and could be due to aberrant connections in collaterals between MSNs or, alternatively, due to hyperactivity of parvalbumin interneurons [86–89]. We postulate however, that the major responsibility for the significant improvement of the R6/1 phenotype, such as this restoration of striatal GABA and glutamate signaling, are thalamo-striatal connections targeting MSNs. Reinforcing this idea we observed a complete rescue of their soma size, their dendritic spine density as well as their DARPP-32 levels. In contrast, ChAT and PV interneurons were altered in R6/1 controls without apparent improvements in R6/1 with restoration of thalamic Foxp2 levels.

On the other hand, aberrant increases in GABA concentration and signaling in motor brain regions have been associated with motor disturbances in the context of other neurological disorders [90, 91] and in the context of Foxp2 loss-of-function paradigms [25, 29, 30, 92] suggesting a potential activation/inhibition imbalance in R6/1 mice since striatal glutamate levels are changed to a lesser extent. On the other hand, it is worth mentioning that such GABA activity could modulate oscillatory rhythms as we already observed in non-pathological conditions [93]. Adaptation mechanisms as well as synaptic inhibition mediated by GABA receptors have been proposed as mechanisms governing the termination of Up states, which are altered in R6/1 mice. Such patterns of active and silent cortical activity depend on the balance between recurrent excitation and local inhibition [94–96] which is probably what we observed in R6/1 mice. Finally, models of Foxp2 disruption have also been described to induce striatal excitatory/inhibitory imbalances [30, 32, 92, 97].

In the present study, some questions remain open. First, it would be interesting to test whether thalamo-striatal and thalamo-cortical aberrant and dysfunctional connectivity starts much before than the ages evaluated here. Second, it would be insightful to test from a potential therapeutic point of view how durable in time are our Foxp2 manipulations in rescue experiments. Taken altogether, we conclude that the following sequence: Early reduced thalamic Foxp2 levels >  >  > induction of striatal GABA overproduction and release >  >  > alterations of oscillatory rhythms >  >  > sensorial and motor deficits, could play a pivotal role in the pathophysiology of HD.

### Supplementary Information

Below is the link to the electronic supplementary material.

Supplementary file1 (DOCX 2645 KB)

Supplementary file2 (XLSX 64 KB)

Supplementary file3 (PPTX 30634 KB)

## Data Availability

All data supporting the findings of this study are available within the paper and its Supplementary Information or it is available from the authors upon reasonable request.
