# Supplementary material for: Thalamic Foxp2 regulates output connectivity and sensory-motor impairments in a model of Huntington’s Disease
Source: Cell Mol Life Sci. 2023 Nov 21;80(12):367. doi: 10.1007/s00018-023-05015-z (PMC10663254; doi:10.1007/s00018-023-05015-z)
Supplement: Supplementary file 1 — Supplementary file1 (DOCX 2645 KB) [file 18_2023_5015_MOESM1_ESM.docx]

**Supplementary figures and tables**

**Supplementary table 1. Baseline Glu and GABA dialysate concentrations in CPu of mice.**

| **Experimental groups** | **Baseline Glu levels** | **Baseline GABA levels** |
| --- | --- | --- |
| WT-GFP | 2.5 ± 0.4 (6) | 14.5 ± 2.7 (6) |
| WT-Foxp2 | 2.5 ± 0.6 (5) | 14.7 ± 2.8 (5) |
| R6/1-GFP | 2.2 ± 0.3 (6) | 11.3 ± 1.9 (6) |
| R6/1-Foxp2 | 2.3 ± 0.4 (5) | 17.9 ± 1.6 (5) |

Extracellular Glu and GABA levels are expressed as pmol/20‐min fraction or fmol/20-fraction, respectively. Data are the mean ± SEM. The number of mice used in each experimental group is indicated in parentheses.

**Supplementary figure 1. Schematic location and distribution of the injection sites for the rabies-based studies.**

**
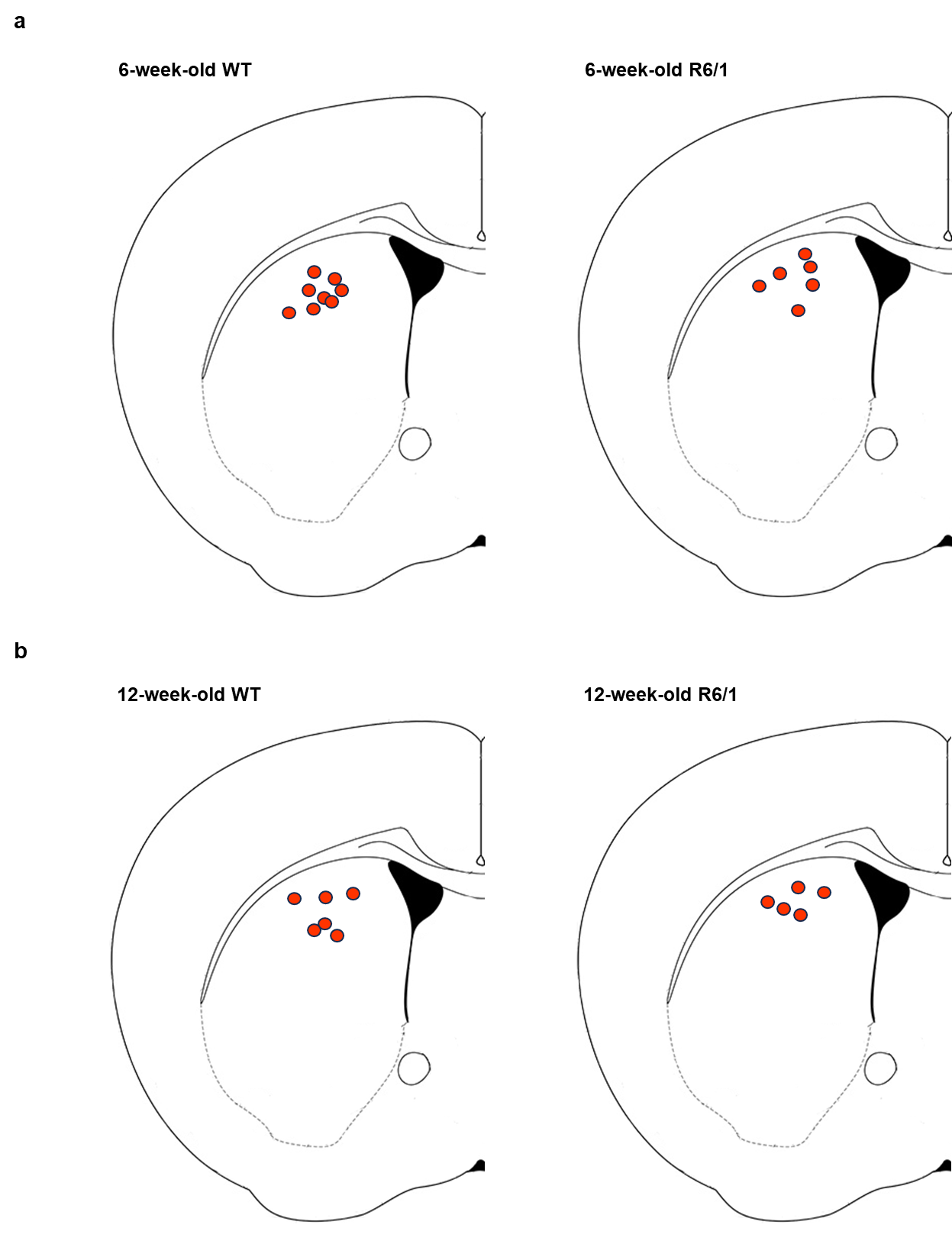
**

**a** Schematic location and distribution of the injection sites in dorsal striatum of 6-week-old WT (left panel) and R6/1 (right panel) mice. Red dots indicate the center of the injections for each mouse used in the study. **b** Schematic location and distribution of the injection sites in dorsal striatum in 12-week-old WT (left panel) and R6/1 (right panel) mice. Red dots indicate the center of the injections for each mouse used in the study. The location in **a** and **b** is based on the identification of the pseudo-typed rabies virus EnvA-ΔG-mCherry labeling.

**Supplementary figure 2. Characterization of neuronal subpopulations in the striatum of WT and R6/1 mice transduced with AAV-GFP or AAV-Foxp2.**

**
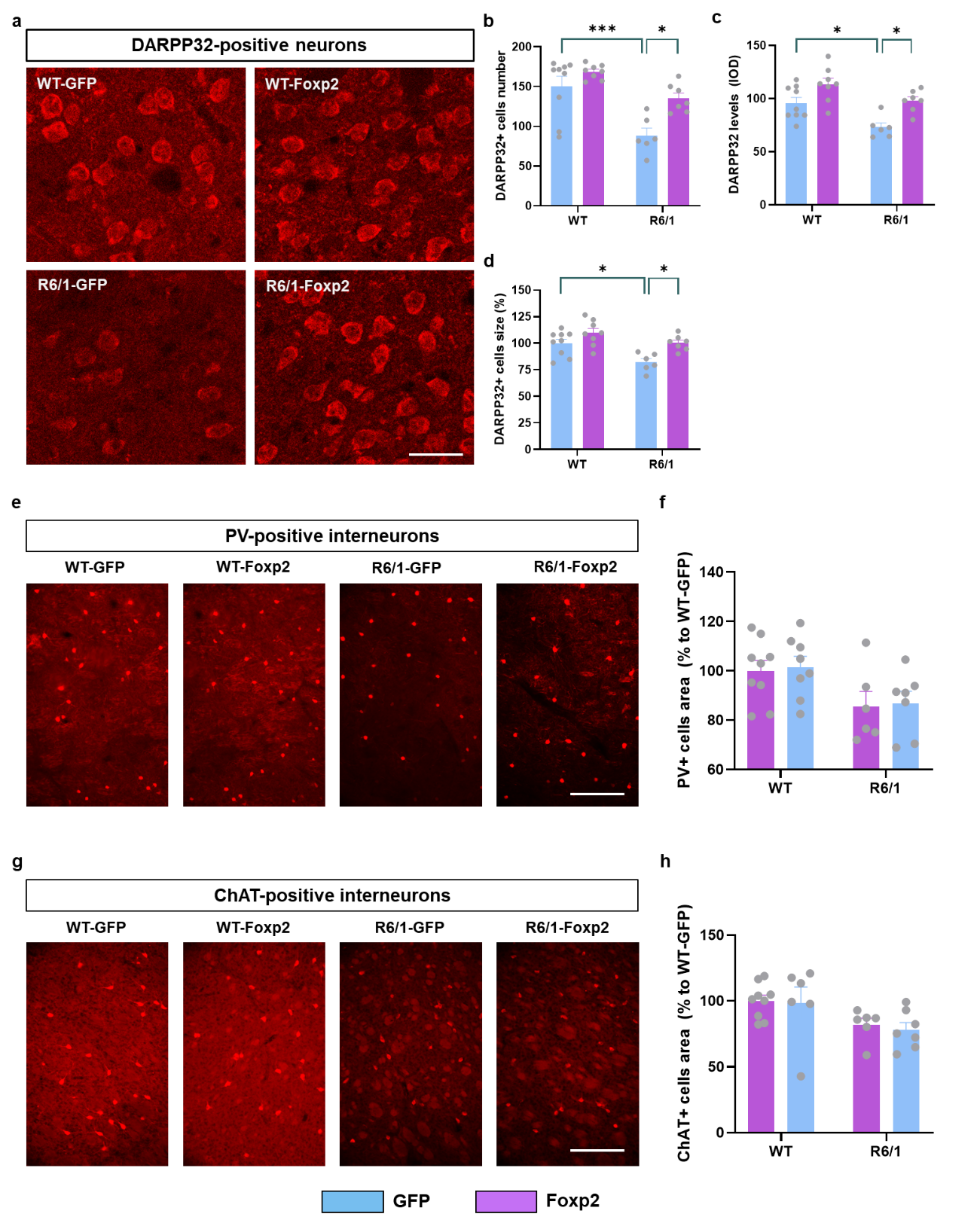
**

**a** Representative microphotograph of coronal sections from WT-GFP, WT-Foxp2, R6/1-GFP and R6/1-Foxp2 groups of mice immunostained for DARPP-32 in the dorsal striatum. Mouse samples come from the experiment depicted in figure 3. **b** The number of DARPP-32-positive projecting neurons/field was estimated in all four groups of mice. For each mouse, two images were taken in the dorsal striatum per slice and in two different slices. From the same images as in **b** we also calculated in **c** the DARPP-32 integrated optical density (IOD) and in **d** the size (µm^2^ relativized to the WT-GFP group) of DARPP-32-positive cells. In **b**, two-way ANOVA, genotype effect: F(1,26) = 27.26; p < 0.0001; treatment effect: F(1,26) = 12.46; p = 0.0016. In **c**, two-way ANOVA, genotype effect: F(1,26) = 14.46; p = 0.0008; treatment effect: F(1,26) = 17.29; p = 0.0003. In **d**, two-way ANOVA, genotype effect: F(1,26) = 12.72; p = 0.0014; treatment effect: F(1,26) = 13.32; p = 0.0012. **e** Representative microphotograph of coronal sections from WT-GFP, WT-Foxp2, R6/1-GFP and R6/1-Foxp2 groups of mice immunostained for parvalbumin (PV) in the striatum. Mouse samples come from the experiment depicted in figure 3. **f** The area (number of positive pixels) occupied by PV-positive neurons/striatum was estimated in all four groups of mice. For each mouse, two mosaics (from two different slices) of the entire striatum were taken. In **f** two-way ANOVA, genotype effect: F(1,26) = 9.025; p = 0.0058. **g** Representative microphotograph of coronal sections from WT-GFP, WT-Foxp2, R6/1-GFP and R6/1-Foxp2 groups of mice immunostained for Choline acetyltransferase (ChAT) in the striatum. Mouse samples come from the experiment depicted in figure 3. **h** The area (number of positive pixels) occupied by ChAT-positive neurons/striatum was estimated in all four groups of mice. For each mouse, two mosaics (from two different slices) of the entire striatum were taken. In **h** two-way ANOVA, genotype effect: F(1,24) = 8.028; p = 0.0092. Scale bar in **a** 30 µm; in **e** and **g** 150 µm. Data are represented as mean ± SEM. Tukey *post hoc* test was employed in **b-c** and in **f** an **h**. * p < 0.05 and *** p < 0.001. WT-GFP (n = 9), WT-Foxp2 (n = 8), R6/1-GFP (n = 6) and R6/1-Foxp2 (n = 7).

**Supplementary figure 3. Characterization of dendritic spine morphologies in the medium spiny and stellate neurons of WT and R6/1 mice transduced with AAV-GFP or AAV-Foxp2.**

**
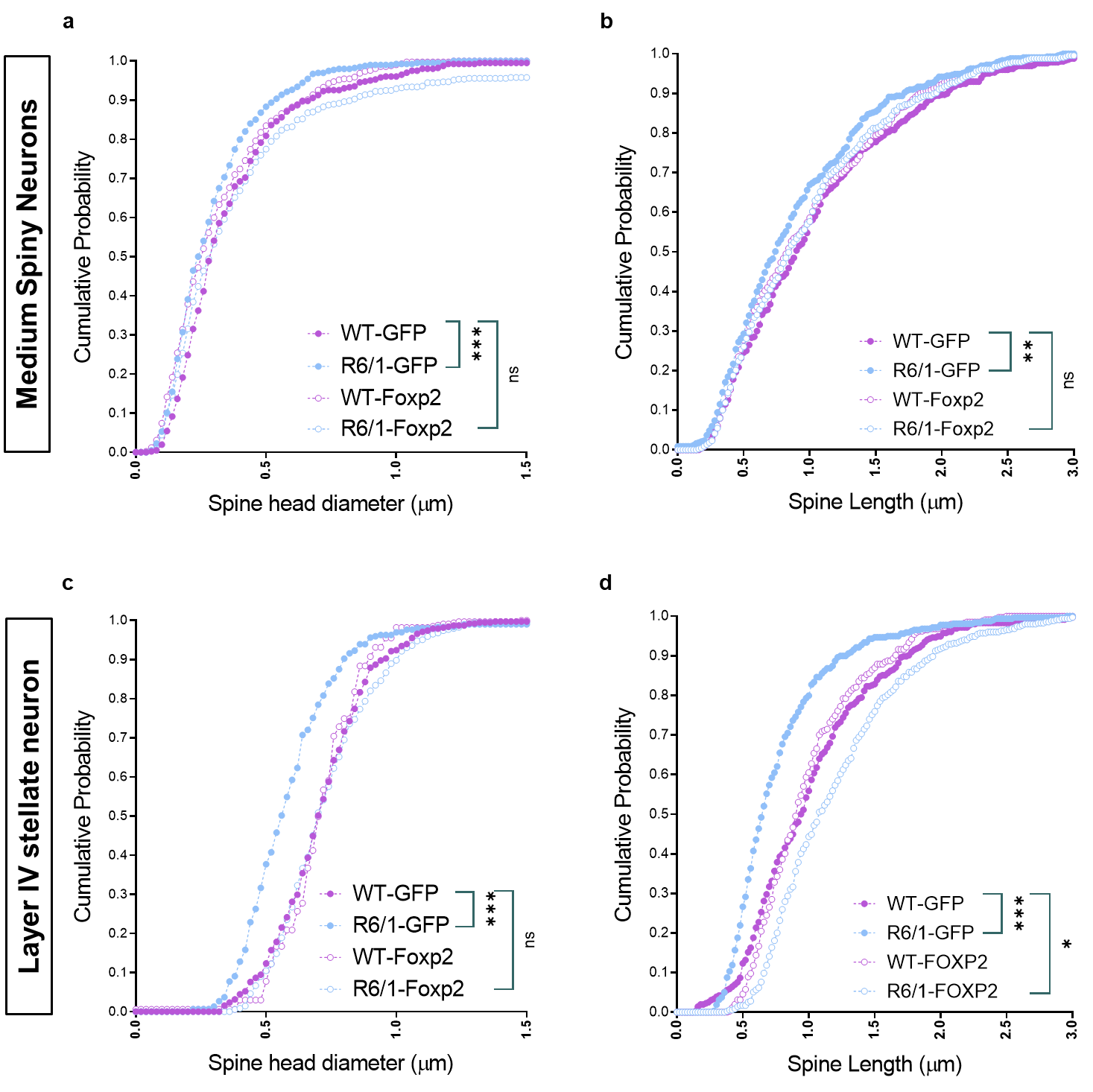
**

**a** Cumulative probability of spine head diameter (n = 394-453) and **b** spine length (n = 427-505) in ∼80 dendrites from medium spiny neurons of the dorsal striatum. Analysis was performed in Golgi-stained samples from figure 4. Four-six animals per group were employed. Distributions were compared by paired comparisons with the Kolmogorov–Smirnov test. Significant differences in spine head diameter distribution were observed in R6/1-GFP (D=0.15338, p < 0.0001) but not in R6/1-Foxp2 when compared with WT-GFP mice. Also, significant differences in spine length distribution were observed in R6/1-GFP (D=0.1154, p = 0.0065) but not in R6/1-Foxp2 when compared with WT-GFP mice. **c** Cumulative probability of spine head diameter (n = 297-381) and **d** spine length (n = 319-356) in ∼80 dendrites from stellate neurons of the motor cortex. Significant differences in spine head diameter distribution were observed in R6/1-GFP (D=0.3708, p < 0.0001) but not in R6/1-Foxp2 when compared with WT-GFP mice. Also, significant differences in spine length distribution were observed in R6/1-GFP (D=0.2914, p < 0.0001) when compared with WT-GFP mice. In contrast, R6/1-Foxp2 mice also displayed a significantly different spine length distribution (D=0.1878, p < 0.0001) compared to WT-GFP mice but in an opposite way regarding to that observed in R6/1-GFP mice.

**Supplementary figure 4. Characterization of spontaneous neural activity and thalamic evoked responses in motor and somatosensory cortices in R6/1 mice.**


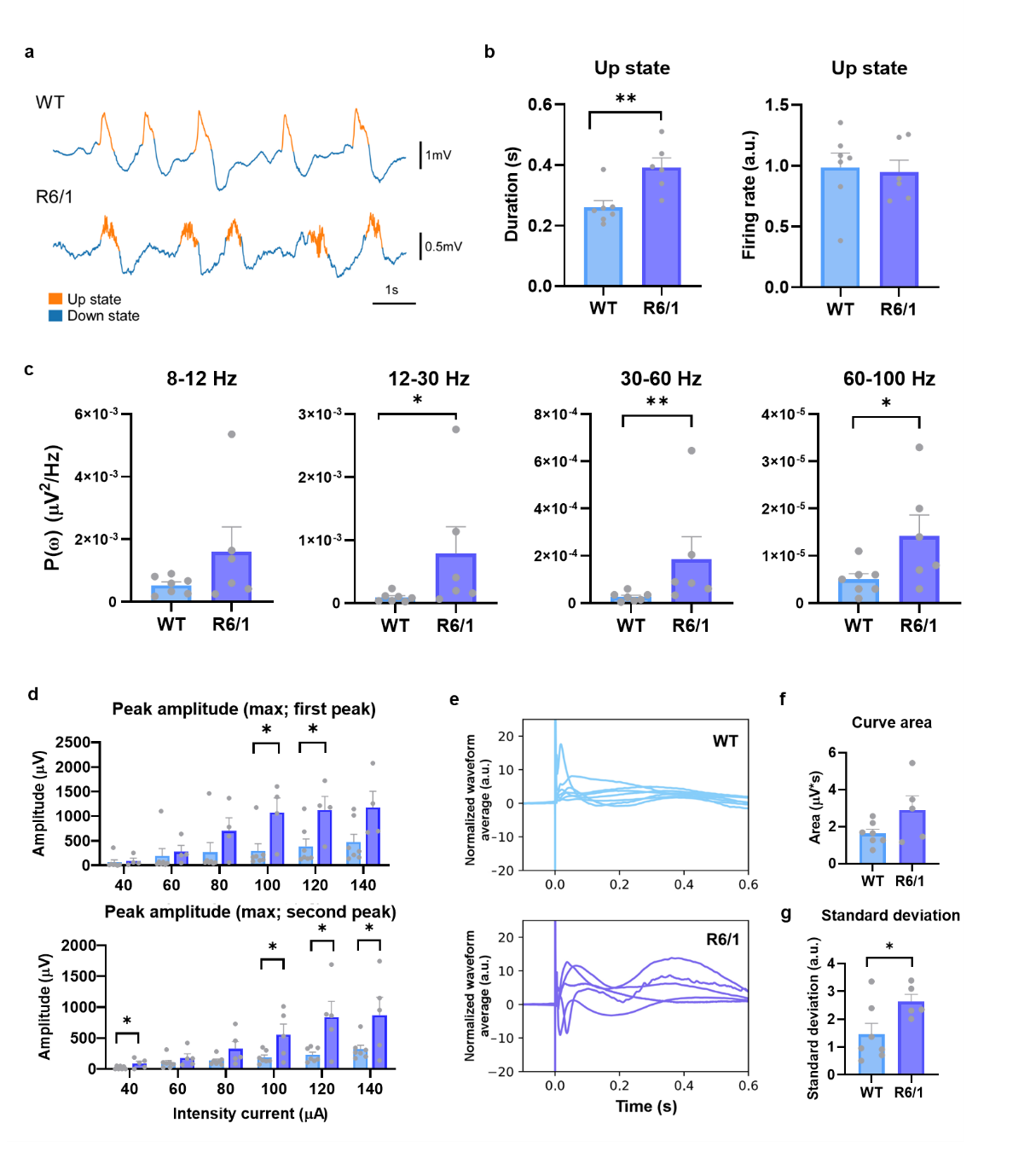


**a** Spontaneous local field potentials (LFP) were recorded during slow oscillation activity in deeply anesthetized WT and R6/1 mice from different motor and somatosensory cortical areas thought a superficial 32-channels multi-electrode array placed in the cortex. Representative raw traces examples of the LFP are shown for each experimental group. Up and down events are colored in orange and blue, respectively. **b** Quantification of the Up states mean duration (in seconds; left panel) and the mean firing rate (in arbitrary units, a.u.; right panel) during the Up states in WT and R6/1 mice. **c** Averaged power spectral density (PSD) over the z-scored normalized LFP of oscillatory activity at different frequency bands (alpha, 8-12 Hz; beta, 12-30 Hz; low-gamma, 30-60 Hz; high-gamma, 60-100 Hz) in WT and R6/1 mice. **d** Evoked responses were elicited in the cortex by applying 50 electrical stimulation pulses (0.1 Hz, 0.3 ms) with an intensity current range of 20-160 µA in the ventrobasal thalamus. These responses in a 50 ms time-window generally consist of a first positive wave that are followed by a second negative wave. The responses evoked in the 32 channels were quantified, and the maximum peak amplitude of the first (upper panel) and the second (lower panel) waves of the responses are shown for the range of 40-140 µA intensity currents. **e** Long-lasting baseline z-score normalized evoked responses (in a.u.) after thalamic stimulation at 160 µA in WT (blue; upper panel) and R6/ 1 mice (purple; lower panel). Solid lines indicate the trial-averaged waveforms of each animal. **f** The area under the curve were quantified for a time-window of 600 ms post-stimuli using the 160 µA intensity current protocol for each experimental group. **g** The value distribution of the area under the curve of the normalized evoked responses in the 32 channels recorded was quantified as the standard deviation (in a.u.) for WT and R6/1 mice. Data are represented as mean ± SEM. Mann-Withney test was used **b** and **c**. Unpaired t-test was used in **d**, **f** and **g**, * p < 0.05, ** p < 0.01 compared with WT mice.

**Supplementary figure 5. Characterization of neuronal subpopulations in the striatum of WT mice transduced with AAV-Scramble or AAV-shFoxp2.**

**
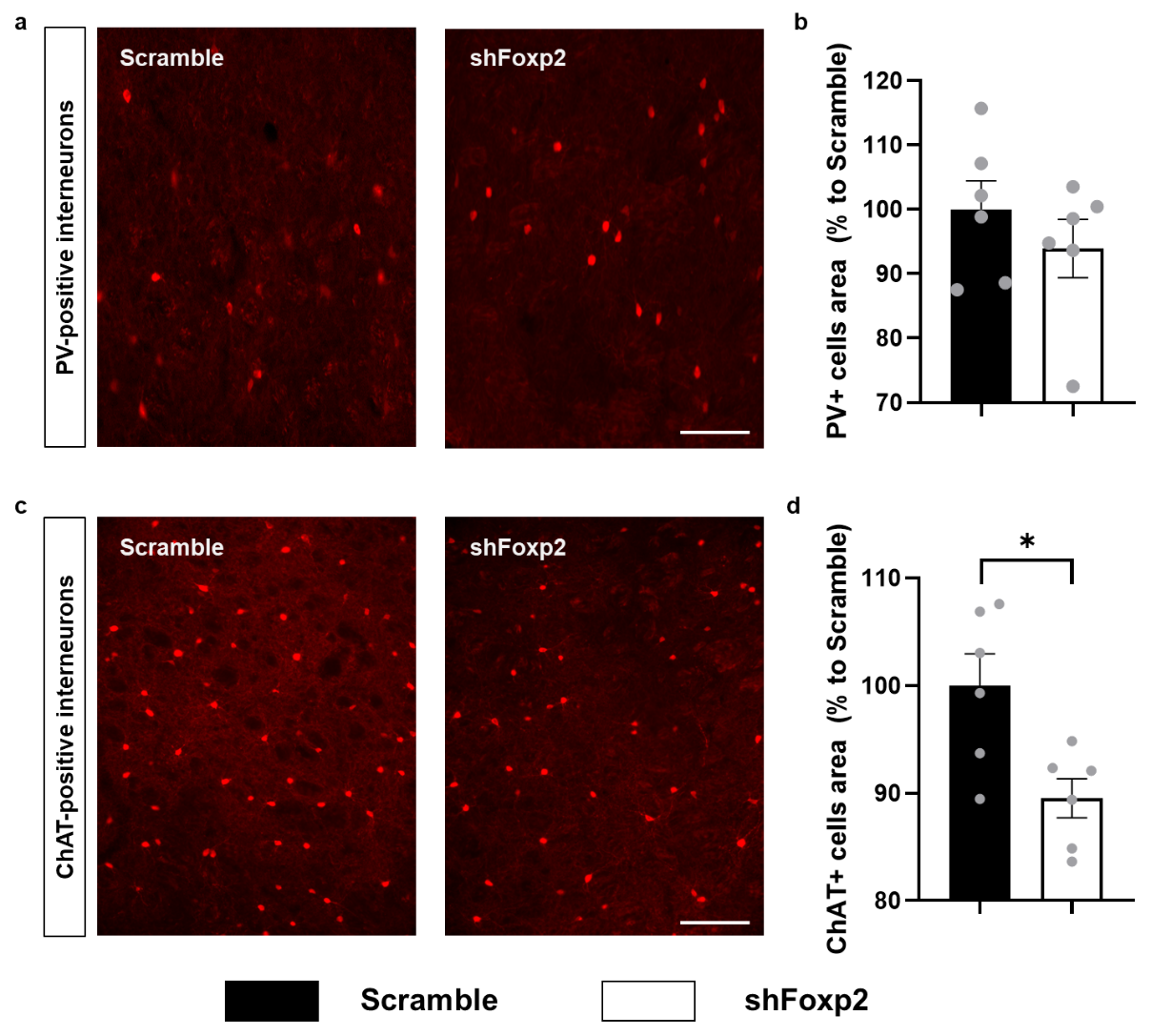
**

**a** Representative microphotograph of coronal sections from Scramble and shFoxp2 groups of mice immunostained for parvalbumin (PV) in the striatum. Mouse samples come from the experiment depicted in figure 8. **b** The area (number of positive pixels) occupied by PV-positive neurons/striatum was estimated in both groups of mice. For each mouse, two mosaics (from two different slices) of the entire striatum were taken. **c** Representative microphotograph of coronal sections from Scramble and shFoxp2 groups of mice immunostained for Choline acetyltransferase (ChAT) in the striatum. Mouse samples come from the experiment depicted in figure 8. **d** The area (number of positive pixels) occupied by ChAT-positive neurons/striatum was estimated in both groups of mice. For each mouse, two mosaics (from two different slices) of the entire striatum were taken. Unpaired t-test: t=3,007, df=10, p = 0.0132. Scale bar in **a** and **c** 100 microns. Data are represented as mean ± SEM. Scramble (n = 6), shFoxp2 (n = 6).
