# Supplementary material for: Thalamic Foxp2 regulates output connectivity and sensory-motor impairments in a model of Huntington’s Disease
Source: Cell Mol Life Sci. 2023 Nov 21;80(12):367. doi: 10.1007/s00018-023-05015-z (PMC10663254; doi:10.1007/s00018-023-05015-z)
Supplement: Supplementary file 3 — Supplementary file3 (PPTX 30634 KB) [file 18_2023_5015_MOESM3_ESM.pptx]

## Slide 1
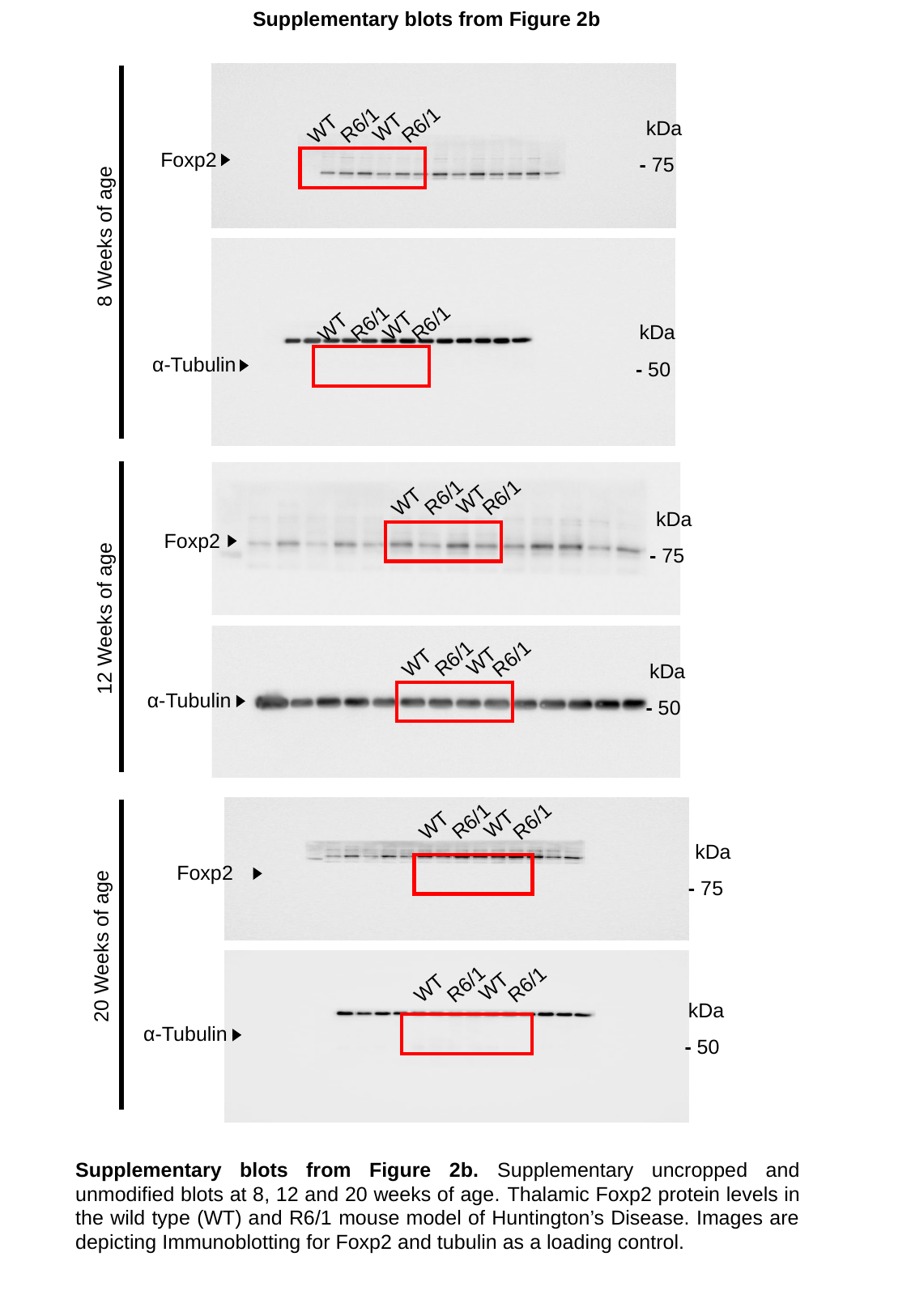

Supplementary blots from Figure 2b
R6/1
R6/1
WT
WT
kDa
Foxp2
- 75
8 Weeks of age
R6/1
R6/1
WT
WT
kDa
α-Tubulin
- 50
R6/1
R6/1
WT
WT
kDa
Foxp2
- 75
12 Weeks of age
R6/1
R6/1
WT
WT
kDa
α-Tubulin
- 50
R6/1
R6/1
WT
WT
kDa
Foxp2
- 75
20 Weeks of age
R6/1
R6/1
WT
WT
kDa
α-Tubulin
- 50
Supplementary blots from Figure 2b. Supplementary uncropped and unmodified blots at 8, 12 and 20 weeks of age. Thalamic Foxp2 protein levels in the wild type (WT) and R6/1 mouse model of Huntington’s Disease. Images are depicting Immunoblotting for Foxp2 and tubulin as a loading control.

## Slide 2
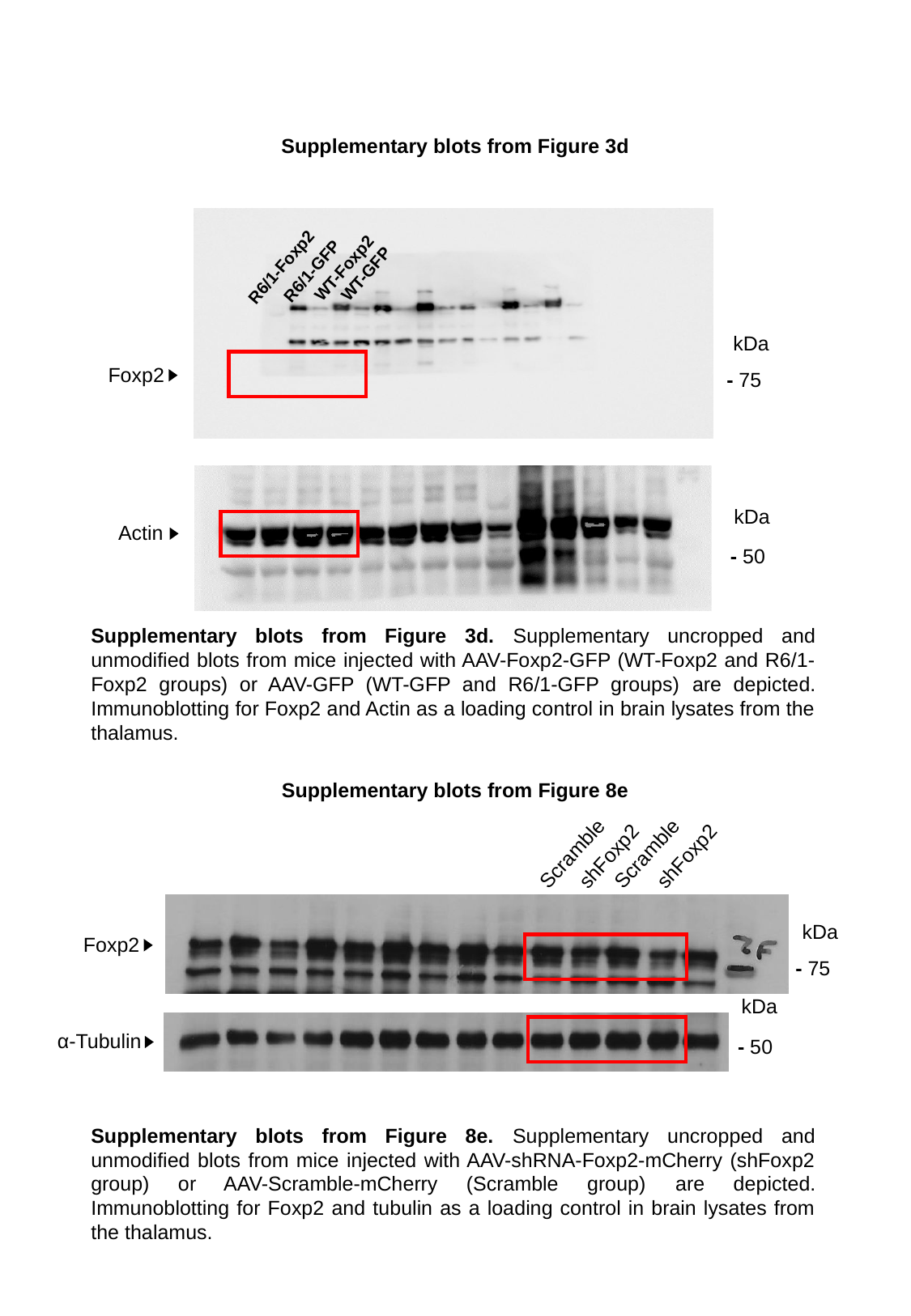

Supplementary blots from Figure 3d
R6/1-Foxp2
R6/1-GFP
WT-GFP
WT-Foxp2
kDa
Foxp2
- 75
kDa
Actin
- 50
Supplementary blots from Figure 3d. Supplementary uncropped and unmodified blots from mice injected with AAV-Foxp2-GFP (WT-Foxp2 and R6/1-Foxp2 groups) or AAV-GFP (WT-GFP and R6/1-GFP groups) are depicted. Immunoblotting for Foxp2 and Actin as a loading control in brain lysates from the thalamus.
Supplementary blots from Figure 8e
Scramble
shFoxp2
Scramble
shFoxp2
kDa
Foxp2
- 75
kDa
α-Tubulin
- 50
Supplementary blots from Figure 8e. Supplementary uncropped and unmodified blots from mice injected with AAV-shRNA-Foxp2-mCherry (shFoxp2 group) or AAV-Scramble-mCherry (Scramble group) are depicted. Immunoblotting for Foxp2 and tubulin as a loading control in brain lysates from the thalamus.
